# A mixed-modeling framework for whole-brain dynamic network analysis

**DOI:** 10.1162/netn_a_00238

**Published:** 2022-06-01

**Authors:** Mohsen Bahrami, Paul J. Laurienti, Heather M. Shappell, Dale Dagenbach, Sean L. Simpson

**Affiliations:** Laboratory for Complex Brain Networks, Wake Forest School of Medicine, Winston-Salem, NC, USA; Department of Radiology, Wake Forest School of Medicine, Winston-Salem, NC, USA; Department of Biostatistics and Data Science, Wake Forest School of Medicine, Winston-Salem, NC, USA; Department of Psychology, Wake Forest University, Winston-Salem, NC, USA

**Keywords:** Mixed models, fMRI, Dynamic brain networks, Multivariate, Simulation

## Abstract

The emerging area of dynamic brain network analysis has gained considerable attention in recent years. However, development of multivariate statistical frameworks that allow for examining the associations between phenotypic traits and dynamic patterns of system-level properties of the brain, and drawing statistical inference about such associations, has largely lagged behind. To address this need we developed a mixed-modeling framework that allows for assessing the relationship between any desired phenotype and dynamic patterns of whole-brain connectivity and topology. This novel framework also allows for simulating dynamic brain networks with respect to desired covariates. Unlike current tools, which largely use data-driven methods, our model-based method enables aligning neuroscientific hypotheses with the analytic approach. We demonstrate the utility of this model in identifying the relationship between fluid intelligence and dynamic brain networks by using resting-state fMRI (rfMRI) data from 200 participants in the Human Connectome Project (HCP) study. We also demonstrate the utility of this model to simulate dynamic brain networks at both group and individual levels. To our knowledge, this approach provides the first model-based statistical method for examining dynamic patterns of system-level properties of the brain and their relationships to phenotypic traits as well as simulating dynamic brain networks.

## Author Summary

In recent years, a growing body of studies have aimed at analyzing the brain as a complex dynamic system by using various neuroimaging data. This has opened new avenues to answer compelling questions about the brain function in health and disease. However, methods that allow for providing statistical inference about how the complex interactions of the brain are associated with desired phenotypes are to be developed for a more profound insight. This study introduces a promising regression-based model to relate dynamic brain networks to desired phenotypes and provide statistical inference. Moreover, it can be used for simulating dynamic brain networks with respect to desired phenotypes at the group and individual levels.

## INTRODUCTION

The past two decades have witnessed an explosion of studies aimed at examining the brain as a complex system through analysis of neuroimaging data, particularly data from functional MRI (fMRI). Complex functional systems of the brain are often analyzed through graph theoretical measures of the brain’s functional networks (Bullmore & Sporns, 2009). Nodes in brain networks often represent brain regions, and edges represent functional connections (statistical associations) between the blood oxygen level–dependent (BOLD) signals in different brain regions. Until recent years, most network studies of the brain focused on static functional networks, in which the functional connections between brain regions were defined over the entire scanning period or condition. Although such studies have provided promising insights into functional organization and abnormalities of the brain (Bassett & Bullmore, 2009; Park & Friston, 2013), recent studies indicate that functional connectivity patterns are not stationary and fluctuate over very short periods of time on the order of seconds (Chang & Glover, 2010; Handwerker, Roopchansingh, Gonzalez-Castillo, & Bandettini, 2012; Hutchison et al., 2013; Parr, Rees, & Friston, 2018). This has resulted in a new and rapidly evolving line of studies examining dynamic networks or time-varying functional connectivity patterns of the brain.

Studies of brain dynamics are critical for establishing a profound understanding of the brain given that the brain is a complex multiscale dynamic system rather than a stationary one (Lurie et al., 2020). As noted in Breakspear (2017), analytical frameworks that allow for examining the dynamical systems of the brain, from differential equations and state space analyses of populations of neurons to larger scale network science methods, are essential for understanding how behavioral and cognitive processes emerge from neural activities. Dynamic brain networks have been associated with a wide range of cognitive and behavioral responses (Cole, Bassett, Power, Braver, & Petersen, 2014; Shine et al., 2016; Vidaurre, Abeysuriya, et al., 2018). More specifically, they have been used to determine the engagement of a participant in a specific cognitive task (Gonzalez-Castillo et al., 2015; Shirer, Ryali, Rykhlevskaia, Menon, & Greicius, 2012), and have been associated with consciousness (Barttfeld et al., 2015; Godwin, Barry, & Marois, 2015), learning (Bassett et al., 2011), and various neuropsychiatric and neurological disorders, such as schizophrenia (Rashid et al., 2016; Sakoglu et al., 2010), depression (Long et al., 2020; Martinez, Deco, Ter Horst, & Cabral, 2020), Alzheimer’s disease (Gu et al., 2020; Jones et al., 2012), and Parkinson’s disease (Diez-Cirarda et al., 2018; Zhu et al., 2019). New studies indicate that dynamic brain networks may provide more sensitive biomarkers for detecting differences between study populations or individuals than static networks (Rashid et al., 2016). For example, dynamic patterns of brain connectivity at rest have been shown to better characterize people with post-traumatic stress disorder (Jin et al., 2017) or predict weight loss among older adults (Mokhtari, Laurienti, Rejeski, & Ballard, 2019).

Despite such insights, substantial challenges remain to enable more accurate analysis of dynamic brain networks and accurate interpretation of results. Dynamic changes in the systemic organization of brain networks confers much of our brains’ functional abilities (Bressler & Menon, 2010; Buzsaki & Draguhn, 2004). If functional connections are lost or rendered dynamically rigid due to an adverse health condition, compensatory connections may develop to maintain organizational consistency and functional abilities. Consequently, brain network analysis necessitates a suite of tools including a multivariate modeling framework for dynamic brain network data to assess effects of multiple variables of interest and topological network features on the overall network structure. However, most current methods rudimentarily compare the dynamic patterns of connection strength or networks measures across study populations (Elton & Gao, 2015; Fukushima et al., 2018; Sizemore & Bassett, 2018), failing to fully harness the wealth of information about dynamics of system-level properties of the brain, which can be obtained via a validated multivariate statistical method.

To date, despite having a broad range, most analytical approaches have aimed at identifying similar FC patterns (e.g., through clustering methods; Allen et al., 2014) or hidden brain states (often through hidden Markov models; Shappell, Caffo, Pekar, & Lindquist, 2019; Vidaurre, Hunt, et al., 2018) with respect to desired outcomes. Although such methods have provided useful insights, there remain many gaps in the suite of analytic methods available, including the following. (1) One gap is the lack of a well-defined statistical framework to align the neuroscientific hypothesis with the analytical approach and to assess the effects of multiple phenotypes of interest simultaneously. As pointed out earlier, most current methods use clustering-based approaches on FC patterns or hidden Markov models on time series to identify brain states and their transitions with respect to behavioral and cognitive outcomes. Such data-driven methods have allowed for identifying complex patterns of dynamic changes in the brain, providing profound insight into dynamic brain networks. Thus, they may still be the most appropriate methods for studies aimed at examining state-based changes in dynamic brain networks or for comparing study populations in the absence of a well-defined hypothesis. However, to conduct statistical inference (i.e., hypothesis testing) on a well-posited neuroscientific hypothesis about dynamic brain networks, a model-based method that allows selectively incorporating the desired topological properties of the brain or FC patterns as well as combinations of desired variables, will provide a more fruitful framework. Other gaps in the suite of analytic methods include (2) difficulty in controlling for confounding effects, such as age and gender (this often requires matching the study populations, which is a daunting task for most neuroimaging studies); (3) the limitation of examining fluctuations of single network measures instead of capturing the complex dynamics of brain networks as a system; and (4) inability to simulate dynamic brain networks with respect to changes in system-level properties of the brain and desired covariates. Such critical methodological gaps may be addressed through parsimonious multivariate statistical frameworks.

As noted by Shine et al. (2019), the neurobiological mechanisms underlying brain network dynamics (dynamic changes in functional architecture) remain poorly understood; and as pointed out in Liu (2017), “novel methods are urgently needed for a better quantification of temporal dynamics in resting-state fMRI.” The development of rigorous statistical methods within a multivariate framework are among such urgent needs. Developing and disseminating explainable, validated multivariate statistical methods are paramount for relating phenotypic traits to dynamic changes in network properties of the brain, which will greatly aid in providing profound insights into normal and abnormal brain function.

**Figure 1.**
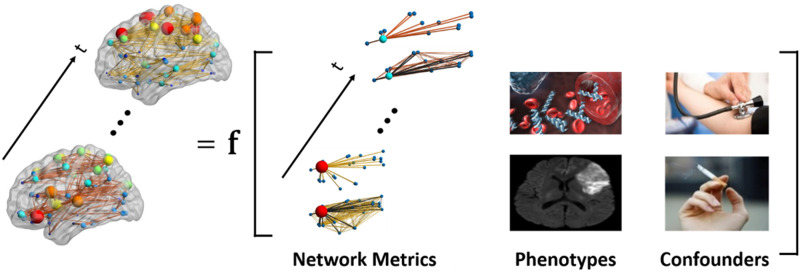
Dynamic brain networks as a function of endogenous and exogenous variables of interest. Dynamic patterns of brain connectivity (presence/absence and strength) is modeled as a function of (dynamic) nodal and global network metrics (e.g., clustering coefficient, global efficiency, etc.) and exogenous covariates, including phenotypes (e.g., blood measurements and brain damage) and possible confounding effects (e.g., hypertension and smoking).

For the modeling framework, if we have

$$\text{Data}\left\{\begin{array}{c} Y_{it}:\text{network}\;\text{of}\;\text{participant}\;i\;\text{at}\;\text{time}\;\text{point}\;t\\ X_{it}:\text{covariate}\;\text{information} \end{array}\right.$$

we wish to accurately estimate the probability density function of the networks given the covariates *P*(***Y***_*it*_|***X***_*it*_, ***β***_*it*_), where ***β***_*it*_ are the parameters that relate the covariates to the network structure as shown in Figure 1. However, the development of such methods has vastly lagged behind, mainly due to the same challenges which exist in developing multivariate statistical tools for static networks (Bahrami, Laurienti, & Simpson, 2019a; Shehzad et al., 2014; Simpson & Laurienti, 2016). Here we introduce a novel multivariate statistical framework for assessing phenotype-dynamic brain network pattern relationships and drawing inference from such relationships.

This *model-based* framework is a fundamentally different approach toward analyzing dynamic brain networks when compared to current approaches, which often use *data-driven* methodologies to identify “brain states” and their transitions with respect to health and behavioral outcomes (Allen et al., 2014; Shappell et al., 2019; Shine & Poldrack, 2018; Vidaurre, Smith, & Woolrich, 2017). We develop this method by advancing a promising statistical mixed-modeling framework for static networks (Simpson & Laurienti, 2015). Several extensions of the original framework (Bahrami et al., 2019a; Simpson, Bahrami, & Laurienti, 2019), as well as a MATLAB toolbox (Bahrami, Laurienti, & Simpson, 2019b) have recently been introduced. However, it has yet to be extended to the dynamic network context. To our knowledge, this proposed extension will be the first to allow relating group- and individual-level characteristics to time-varying changes in spatial and topological brain network properties while also maintaining the capabilities of the original model, such as accounting for variance associated with confounders.

We will demonstrate the utility of this framework by identifying the relationship between fluid intelligence and dynamic brain network patterns by using data from 200 participants from the Human Connectome Project (HCP) (Van Essen et al., 2013) study. Fluid intelligence (gF) refers to reasoning ability and the capacity of an individual to discern patterns or solve problems when that individual doesn’t have or has minimal resources or acquired knowledge to act upon (Cattell, 1987). Understanding the neurobiological underpinnings of gF is of great interest, as it has been associated with a variety of cognitive abilities (Colom & Flores-Mendoza, 2007; Unsworth, Fukuda, Awh, & Vogel, 2014; Ye et al., 2019). We also demonstrate the utility of this new framework for simulating dynamic brain networks, which can be critical for a better understanding of topological variability in time and across individuals with respect to desired covariates. To our knowledge, this simulation capability is also the first to enable simulating representative group-level dynamic networks from an array of desired global/local network measures and phenotypic characteristics.

## MATERIALS AND METHODS

### Motivating Data

We used the rich dataset provided by the HCP study (Van Essen et al., 2013) to be able to explore dynamic functional brain network differences in cognitively variable populations as a function of phenotype, while maintaining continuity with previous analyses to contrast and clearly distinguish the novel utilities of our proposed method. We specifically focused on demonstrating the utility of our framework in assessing the relationship between dynamic functional networks and intelligence due to the great interest in identifying such relationship. The HCP data released to date include 1,200 individuals. Of those, 1,113 (606 female; 283 minority) have complete MRI images, cognitive testing, and detailed demographic information. Participants in the HCP were screened to rule out neurological and psychiatric disorders. All data were collected on 3T Siemens MRI scanners located at Washington University or the University of Minnesota, using identical scanning parameters. The HCP performed extensive testing and development to ensure comparable imaging at the two sites. The BOLD-weighted images were collected using the following parameters: TR = 720 ms, TE = 33.1 ms, voxel size 2 mm^3^, 72 slices, and 1,200 volumes. In this study, we selected a subsample comprising 389 individuals with unique family identification numbers that also passed our image processing quality control assessments. For multiple individuals with the same family identification number, one individual was selected randomly. We initially used the entire 389 individuals, but we further reduced this to 200 individuals (randomly chosen from our subsample) after we faced convergence issues in modeling one of the two-part mixed-effect models. We have provided the HCP identification numbers for the 200 individuals used in this study in Supporting Information Appendix S1 (Table S1). The HCP analyses are an exemplar; importantly, our methods can be applied to any network-based neuroimaging study.

### Dynamic Networks Generation

We used minimally preprocessed resting fMRI (rfMRI) data from HCP (Glasser et al., 2013). We used two scans for each individual, the left-to-right (LR) and right-to-left (RL). For each scan, we used ICA-AROMA (Pruim et al., 2015) to correct for any motion artifact in the rfMRI data. A band-pass filter (0.009–0.08 Hz) was applied to each scan. The LR and RL scans for each individual were then concatenated temporally, and transient artifacts were removed using a windowed wavelet transformation. Then a regression was performed with the mean tissue signals (GM, WM, and CSF), the six movement parameters and derivatives, as well as a binary regressor to account for any mean signal differences between the two scan conditions (LR and RL scans). Our quality control process removed 116 individuals from the analysis. QC included manually checking the rfMRI for warping irregularities as well as remaining motion artifact after the above processing steps. For the remaining individuals, among those with the same family identification number, one individual was selected randomly. This provided a final dataset comprising 389 individuals with unique family identification numbers. For all 389 individuals, we divided the brain into 268 regions based on a functional atlas (Shen, Tokoglu, Papademetris, & Constable, 2013) and averaged all time series within each region to create a single time series for that region. We used a continuous wavelet transform to filter artifact resulting from the LR and RL concatenation with a window size of 30 s (covering 15 s from the ending and starting points of LR and RL time series, respectively). We then prewhitened the time series to avoid undesired autocorrelation effects for our regression analyses and as recommended by (Honari, Choe, Pekar, & Lindquist, 2019) for dynamic network analyses by using a sliding window correlation approach.

**Figure 2.**
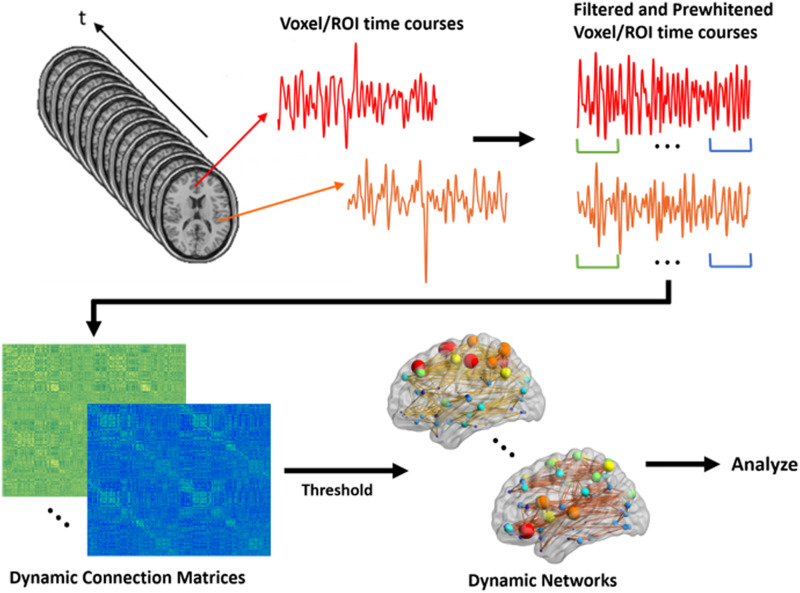
Schematic for generating dynamic brain networks from fMRI time series. Time series are first filtered and prewhitened to remove the undesired undershoots/overshoots in the middle as well as undesired effects of autocorrelation. Then, using a sliding window correlation approach, functional connectivity between brain areas is estimated between all time series pairs at each shift to produce a connection matrix at that shift. By moving the window across the entire length of time series, a series of dynamic connection matrices will be produced for each participant. A threshold is applied to the matrix to remove negative connections. These networks are subsequently used for analyses.

Dynamic brain networks for each participant were constructed through a sliding window correlation approach. We used a modulated rectangular window (Mokhtari, Akhlaghi, Simpson, Wu, & Laurienti, 2019) with a length of 120 volumes and the same shift size (i.e., 120 volumes) to generate windows with no overlap between consecutive networks. We understand that this is not a commonly used shift size as most studies use overlapping windows with 1 TR shift size; however, unlike other methods, we subsequently use the dynamic networks in a regression framework, thus we used windows with no overlap to further reduce temporal autocorrelation. However, in an additional analysis provided in Supporting Information Appendix S2, we used windows with 50% overlap between consecutive networks, that is, using a shift size of 60 volumes, which yielded similar results. Since at least one other study that uses dynamic networks in a regression framework has used windows with 50% overlap between consecutive networks (Chang, Liu, Chen, Liu, & Duyn, 2013), we conducted this additional analysis to see how our reported results would be affected when using this approach. Despite similar results, we present results for windows with no overlap between consecutive networks to ensure that temporal autocorrelation effects are minimized. The reasons and implications of our choices for window type, window length, and shift size will be further explained in the Discussion. The dynamic networks for each participant were generated by moving the window across the time series and computing the Pearson’s correlation between time series of all pairs of 268 regions at each shift. This yielded 19 dynamic networks for each participant. We then thresholded all dynamic networks to remove negative correlations as multiple network measures, particularly clustering, remain poorly understood in networks with negative correlations (Friedman, Landsberg, Owen, Li, & Mukherjee, 2014; Telesford, Simpson, Burdette, Hayasaka, & Laurienti, 2011). Furthermore, distributions of network variables (such as degree) and the neurobiological interpretation of positive and negative edges are different (Parente et al., 2018; A. J. Schwarz & McGonigle, 2011). When including positive and negative edges in an analysis, networks should be generated and assessed separately (A. J. Schwarz & McGonigle, 2011). Figure 2 shows a schematic exhibiting this dynamic network generation process.

We also used the original models (Simpson & Laurienti, 2015) to conduct the same analyses but with static networks to further examine whether/how dynamic and static networks provide different insight into fluid intelligence–brain network associations (see Supporting Information Appendix S3).

### Mixed-Effects Modeling Framework for Weighted Dynamic Brain Networks

Given that we have sparse weighted networks, a two-part mixed-effects model will be employed to model both the probability of a connection (presence/absence) and the strength of a connection, if it exists (Simpson & Laurienti, 2015). The model includes the entire brain connectivity matrix of each participant, endogenous covariates, and exogenous covariates (see Figure 1). The endogenous covariates are summary variables extracted from the network to summarize global topology. The exogenous covariates are the biologically relevant phenotypic variables (e.g., for our data, fluid intelligence, sex, race, and education among others). This statistical framework allows for the evaluation of group and individual effects. Another key feature of the model is the multivariate nature of the statistics. Inclusion of the actual connectivity matrices allows the statistics to be performed on the entire network simultaneously, rather than performing edge-by-edge analyses in a massively univariate fashion.

More specifically, let *Y*_*ijkt*_ represent the *strength* of the connection (quantified as the correlation in our case) and *R*_*ijkt*_ indicate whether a connection is present (*presence* variable) between node *j* and node *k* for the *i*^*th*^ participant at time *t*. Thus, *R*_*ijkt*_ = 0 if *Y*_*ijkt*_ = 0, and *R*_*ijkt*_ = 1 if *Y*_*ijkt*_ > 0 with conditional probabilities:

$$P\left(R_{\text{ijkt}}=r_{\text{ijkt}}|\beta_{r};b_{ri};\gamma_{r};d_{ri}\right)=\left(\begin{array}{l} 1-p_{\text{ijkt}}\left(\beta_{r};b_{ri};\gamma_{r};d_{ri}\right)\;\text{if}\;r_{\text{ijkt}}=0\\ p_{\text{ijkt}}\left(\beta_{r};b_{ri};\gamma_{r};d_{ri}\right)\;\text{if}\;r_{\text{ijkt}}=1 \end{array}\right),$$ (1)

where *p*_*ijkt*_(***β***_*r*_; ***b***_*ri*_; ***γ***_*r*_; ***d***_*ri*_) is the probability of a connection between nodes *j* and *k* for participant *i* at time *t*. We then have the following logistic mixed model (part I model) for the probability of this connection:

$$\text{logit}\left(p_{\text{ijkt}}\left(\beta_{r};b_{ri};\gamma_{r};d_{ri}\right)\right)=X\prime_{\text{ijkt}}\beta_{r}+\left(\sum_{o=1}^{n}\gamma_{ro}s_{\left(o\right)}\left(X_{t}\right)\right)+Z\prime_{\text{ijkt}}b_{ri}+\left(\sum_{o=1}^{n}d_{rio}s_{\left(o\right)}\left(Z_{t}\right)\right),$$ (2)

where ***β***_*r*_ (note that *r* and *s* subscripts simply denote the probability and strength models in Equations 2 and 3, respectively, and have no relationship with *R*_*ijkt*_ or *S*_*ijkt*_ in the next equation) is a vector of population parameters (fixed effects) that relate the probability of a connection to a set of covariates (***X***_*ijkt*_) for each participant and nodal pair (dyad), ***b***_*ri*_ is a vector of participant- and node-specific parameters (random effects) that capture how this relationship varies about the population average (***β***_*r*_) by participant and node (***Z***_*ijkt*_), ***Z***_*ijkt*_ is the design matrix for the random effects, $\sum_{o=1}^{n}$ *γ*_*ro*_*s*_(*o*)_(*X*_*t*_) corresponds to a population-level *n*^*th*^-order orthonormal polynomial model capturing the dynamic trend in the *presence* of connections across time, and $\sum_{o=1}^{n}$ *d*_*rio*_*s*_(*o*)_(*Z*_*t*_) corresponds to an individual-level *n*^th^-order orthonormal polynomial model capturing how much the participant-specific trends deviate from the population trend. *s*_(*o*)_ is the value of the *o*^*th*^ degree polynomial generated from a set of orthonormal polynomials with maximum degree poynomial of *n*, *X*_*t*_ is the design matrix for the population-level orthonormal polynomials at time *t*, and *Z*_*t*_ is the deisgn matrix for the individual-level orthonormal polynomials at time *t* (see Supporting Information Appendix S4 for a simple example of defining the design matrix and orthonormal polynomials). Employing an orthonormal polynomial model in this manner has been shown to accurately represent the trend in time series data while avoiding the computational issues resulting from the use of natural polynomials (Edwards & Simpson, 2014; Simpson & Edwards, 2013). Note that our ***β***_*r*_ and ***β***_*s*_ are population estimates showing if/how the desired covariates are associated with dynamic networks, that is, after accounting for the dynamic trends through incorporating orthonormal polynomials, ***β***_*r*_ and ***β***_*s*_, show if/how the relationships between desired covariates and dynamic networks, on average, are significant.

For the part II model, which aims to model the strength of a connection given that there is one, we let *S*_*ijkt*_ = [*Y*_*ijkt*_|*R*_*ijkt*_ = 1]. In our case, the *S*_*ijkt*_ will be the values of the correlation coefficients between nodes *j* and *k* for participant *i* at time *t*. We can then use Fisher’s Z-transform, denoted as *FZT*, to induce normality for the following mixed model (part II model)

$$FZT\left(S_{\text{ijkt}}\left(\beta_{s};b_{si};\gamma_{s};d_{ri}\right)\right)=X\prime_{\text{ijkt}}\beta_{s}+\left(\sum_{o=1}^{n}\gamma_{so}s_{\left(o\right)}\left(X_{t}\right)\right)+Z\prime_{\text{ijkt}}b_{si}+\left(\sum_{o=1}^{n}d_{sio}s_{\left(o\right)}\left(Z_{t}\right)\right)+e_{\text{ijkt}},$$ (3)

where ***β***_*s*_ is a vector of population parameters that relate the strength of a connection to the same set of covariates (***X***_*ijkt*_) for each participant and nodal pair (dyad), ***b***_*si*_ is a vector of participant- and node-specific parameters that capture how this relationship varies about the population average (***β***_*s*_) by participant and node (***Z***_*ijkt*_), $\sum_{o=1}^{n}$ *γ*_*so*_*s*_(*o*)_(*X*_*t*_) corresponds to a population-level *n*^*th*^-order orthonormal polynomial model capturing the dynamic trend in the *strength* of connections across time, $\sum_{o=1}^{n}$ *d*_*sio*_*s*_(*o*)_(*Z*_*t*_) corresponds to an individual-level *n*^*th*^-order orthonormal polynomial model capturing how much the participant-specific trends deviate from the population trend, and *e*_*ijkt*_ accounts for the random noise in the connection strength of nodes *j* and *k* for participant *i* at time *t*.

In this study, the covariates (***X***_*ijkt*_) used to explain and predict both the presence and strength of connection include the following. (1) *Net*: the average of the following network variables (categorized and further detailed in Table 1 and in Rubinov and Sporns [2010] and Simpson, Bowman, and Laurienti [2013]) in each dyad: Clustering Coeficient (*C*), Global Efficiency (*Eglob*), Degree (*k*) (difference between connected nodes instead of average to capture “assortativity”), Modularity (*Q*), and Leverage Centrality (Joyce, Laurienti, Burdette, & Hayasaka, 2010) (*l*). Clustering coefficient and global efficiency have been widely used as hallmark measures of segregation (presence of highly interconnected regions supporting regional specialization) and integration (widespread connectivity interconnecting specialized regions) in the brain, respectively (Rubinov & Sporns, 2010).

**Table 1.** Network measures by category

**Category**	**Measure(s)**	**Type**
**Functional segregation**	Clustering coefficient	Local measure
**Functional integration**	Global efficiency	Global(/Local) measure
**Resilience**	Degree difference	Local measure
**Centrality and information flow**	Leverage centrality	Local measure
**Community structure**	Modularity	Global measure

We have used degree difference to capture assortativity, which provides a profound characterization of network resilience (Rubinov & Sporns, 2010). Leverage centrality measures the extent of connectivity of a node relative to its neighbors’ connectivity (Joyce, Laurienti, Burdette, & Hayasaka, 2010). This characterizes the importance of each node for information flow in the brain. Modularity is a hallmark measure of community structure in the brain which has been associated with cognitive performance and intelligence as will be discussed in more detail in the Discussion. (2) *COI*: Covariates of Interest (fluid intelligence (gF) in our study – we modeled gF as a continuous covariate to maximize power, however, in separate analysis, we used gF as a median split binary variable (low/high) which yielded similar results. The binary results can be found in the Supporting Information Appendix S5). gF in the HCP protocol has been assessed using the Raven’s progressive matrices with 24 items with scores being integers representing number of correct items (Bilker et al., 2012); (3) *Int*: Interactions of the Covariate of Interest with the variables in 1; and (4) *Con*: Confounders (for our data: Sex (binary), Age (continuous), years of Education (categorical with three levels − level 1 (≤11), level 2 (12–16), and level 3 (≥17)), BMI (continuous), Race (categorical with six categories − cat 1 (Am. Indian/Alaskan Nat.), cat 2 (Asian/Nat. Hawaiian/Other Pacific Is.), cat 3 (Black or African Am.), cat 4 (White), cat 5 (More than one), cat 6 (Unknown or Not Reported)), Ethnicity (categorical with three categories − cat 1 (Hispanic/Latino), cat 2 (Not Hispanic/Latino), cat 3 (Unknown or Not Reported)), Handedness (continuous − ranging from −100 to +100, with negative and positive numbers indicating whether participants were more left- or right-handed, respectively, assessed using the Edinburgh Handedness Inventory (Oldfield, 1971), Income (Continuous − Total household income), Alcohol abuse (Binary − Indicating whether participant met DSM4 criteria for alcohol abuse), Alcohol dependence (Binary − Indicating whether participant met DSM4 criteria for alcohol dependence), Smoking status (Binary − Indicating whether participant smoked or not), Spatial distance between nodes (importance of spatial distance as potential geometric confounders has been discussed in Friedman et al. (2014), and square of spatial distance between nodes). We used these confounding variables given their widely studied effects on intelligence. Thus, we can decompose ***β***_*r*_ and ***β***_*s*_ into ***β***_*r*_ = [*β*_*r*,0_ ***β***_*r*,*net*_ *β*_*r*,*coi*_ ***β***_*r*,*int*_ ***β***_*r*,*con*_] and ***β***_*s*_ = [*β*_*s*,0_ ***β***_*s*,*net*_ *β*_*s*,*coi*_ ***β***_*s*,*int*_ ***β***_*s*,*con*_] to correspond with the population intercepts and these covariates. For the random-effects vectors we have that ***b***_*ri*_ = [*b*_*ri*,0_ ***b***_*ri*,*net*_ ***b***_*ri*,*dist*_ ***δ***_*ri*,*j*_***δ***_*ri*,*k*_] and ***b***_*si*_ = [*b*_*si*,0_ ***b***_*si*,*net*_ ***b***_*si*,*dist*_ ***δ***_*si*,*j*_***δ***_*si*,*k*_], where *b*_*ri*,0_ and *b*_*si*,0_ quantify the deviation of participant-specific intercepts from the population intercepts (*β*_*r*,0_ and *β*_*s*,0_), ***b***_*ri*,*net*_, and ***b***_*si*,*net*_ contain the participant-specific parameters that capture how much the relationships between the network variables in (1) and the *presence* and *strength* of a connection vary about the population relationships (***β***_*r*,*net*_ and ***β***_*s*,*net*_), respectively, ***b***_*ri*,*dist*_ and ***b***_*si*,*dist*_ contain the participant-specific parameters that capture how much the relationship between spatial distance (and square of spatial distance) and the *presence* and *strength* of a connection vary about the population relationships, respectively, ***δ***_*ri*,*j*_ and ***δ***_*si*,*j*_ contain nodal-specific parameters that represent the propensity for node *j* (of the given dyad) to be connected and the magnitude of its connections, respectively, and ***δ***_*ri*,*k*_ and ***δ***_*si*,*k*_ contain nodal-specific parameters that represent the propensity for node *k* (of the given dyad) to be connected and the magnitude of its connections, respectively. Parameters for all 19 time points (number of networks per individual) (*t* = 1, 2, …, 19) are estimated or predicted simultaneously from the model. In general, additional covariates can also be incorporated as guided by the biological context.

Specifying a reasonable covariance model (balancing appropriate complexity with parsimony and computational feasibility) is paramount for a unified dynamic model such as the one developed here. Toward this end, we assume that ***b***_*ri*_, ***d***_*ri*_, ***b***_*si*_, ***d***_*si*_, and ***e***_*i*_ are normally distributed and mutually independent, with variance component covariance structures for ***b***_*ri*_, ***d***_*ri*_, ***b***_*si*_, and ***d***_*si*_, and the standard conditional independence structure for ***e***_*i*_. That is, ***b***_*ri*_ ∼ *N*(**0**, **Σ**_*bri*_(***τ***_*br*_) = *diag* (***τ***_*br*_)), where ***τ***_*br*_ = ($\sigma_{br,0}^{2}$, $\sigma_{br,net}^{2}$, $\sigma_{br,\text{dist}}^{2}$, $\sigma_{br,\text{node}1}^{2}$, $\sigma_{br,\text{node}2}^{2}$, …, $\sigma_{br,\text{node}268}^{2}$)′, ***d***_*ri*_ ∼ *N*(**0**, **Σ**_*dri*_(***τ***_*dr*_) = *diag* (***τ***_*dr*_)), where ***τ***_*dr*_ = ($\sigma_{dr,0}^{2}$, $\sigma_{dr,1}^{2}$, …, $\sigma_{dr,n}^{2}$)′, ***b***_*si*_ ∼ *N*(**0**, **Σ**_*bsi*_(***τ***_*bs*_) = *diag* (***τ***_*bs*_)), where ***τ***_*bs*_ = ($\sigma_{bs,0}^{2}$, $\sigma_{bs,net}^{2}$, $\sigma_{bs,\text{dist}}^{2}$, $\sigma_{bs,\text{node}1}^{2}$, $\sigma_{bs,\text{node}2}^{2}$, …, $\sigma_{bs,\text{node}268}^{2}$)′, and ***d***_*si*_ ∼ *N*(**0**, **Σ**_*dsi*_(***τ***_*ds*_) = *diag* (***τ***_*ds*_)) where ***τ***_*ds*_ = ($\sigma_{ds,0}^{2}$, $\sigma_{ds,1}^{2}$, …, $\sigma_{ds,n}^{2}$)′—yielding (276 + (*n* + 1)) random effects variance parameters for both the presence and strength models—and ***e***_*i*_ ∼ *N*(**0**, **Σ**_*ei*_ = *σ*^2^***I***). These variance and covariance parameters will provide insight into whether individual and group differences in variability in dynamics relate to health and behavioral outcomes. Parameter estimation is conducted via restricted pseudo-likelihood (Wolfinger & Oconnell, 1993) with the residual approximation of the *F* test for a Wald statistic employed for inference.

We implemented the models (Equations 2 and 3) above to describe and compare brain network dynamics as a function of fluid intelligence. For both models, we started model fitting with the entire set of random effects, that is, random effects for intercept, nodal network measures (clustering, global efficiency, degree, and leverage centrality), distance, and nodal propensities. However, after facing convergence issues, we dropped nodal propensities from our random effects. We assessed model goodness-of-fit (GOF) and consistency of estimates (to further avoid overfitting) to determine the orthonormal polynomial degree yielding the best model fits. We fit the two-part model defined above with the mentioned fixed- and random-effect parameters by using orthonormal polynomial models of degrees ranging from 3–18 (giving 16 model fits), and determined the “best” model based on a composite approach employing the Akaike information criterion (AIC) (Akaike, 1981), Bayesian information criterion (Schwarz, 1978), modified AIC (AICc) (Hurvich & Tsai, 1989), Hannan–Quinn information criterion (Hannan & Quinn, 1979), and consistent AIC (CAIC) (Bozdogan, 1987) GOF measures as well as the consistency of the obtained parameter estimates and *p* values to further avoid overfitting. We used MATLAB to generate the appropriate data frame for our framework and used SAS v9.4 on a Linux operating system with 330 GB of RAM and 2.60 GH processor to perform the model fitting. We have provided the essential SAS macro employed in fitting the statistical mixed models for both Equations 2 and 3 in Supporting Information Appendix S6.

### Simulations

We used the fitted models from Equations 2 and 3 to simulate dynamic brain networks. We used covariates from 50 participants (selected randomly from 200) and 10 dynamic networks (selected randomly from 19), and simulated 10 realizations for each dynamic network of each participant. This yielded a total of 5,000 simulated dynamic networks. To simulate each dynamic network, we first simulated the existence of edges (presence/absence) for all 35,778 node pairs (vectorized symmetric network with 268 nodes) from a Bernoulli distribution with the probability from the fitted model (*p*_*ijkt*_(***β***_*r*_; ***b***_*ri*_; ***γ***_*r*_; ***d***_*ri*_) from Equation 2 and the covariates used for each participant’s dynamic network. We simulated the random-effect coefficients (***γ***_*r*_, ***d***_*ri*_) for each participant from a normal distribution with mean zero and the covariance matrix obtained from the estimated parameters for random effects in Equation 2. To simulate the strength values, we first simulated continuous values from a normal distribution with the mean and covariance obtained from the fitted model in Equation 3 and the covariates for each dynamic network (*N*, (*μ*_*sim*_ = $X\prime_{\text{ijkt}}$***β***_*s*_ + ($\sum_{o=1}^{n}$ *γ*_*so*_*s*_(*o*)_(*X*_*t*_)), $\sigma_{sim}^{2}$ = ***Z***_*ijkt*_**Σ**_*bsi*_(***τ***_*bs*_)$Z\prime_{\text{ijkt}}$ + ***Z***_*t*_**Σ**_*dsi*_(***τ***_*ds*_)$Z\prime_{t}$ + *σ*^2^***I***)). We then used the inverse Fisher’s Z-transform to get the untransformed values, and finally multiplied the resulting vector by the simulated binary vector to get the simulated strength values for the weighted network. We then calculated several (weighted) descriptive measures often used in analyzing brain networks to compare the simulated and observed networks with respect to such topological measures as this provides the most appropriate way to assess the GOF of statistical models in the network context (Hunter, Goodreau, & Handcock, 2008). All simulations were done in MATLAB. The MATLAB script is provided in the Supporting Information Appendix S7. We have also provided the HCP identification numbers for the 50 randomly chosen participants used in our simulation along with the numbers of their 10 randomly chosen dynamic networks in Supporting Information Appendix S8.

## RESULTS

Here, we show our framework’s ability in identifying the relationship between fluid intelligence and dynamic brain networks and its utility for simulating dynamic brain networks. For orthonormal polynomial models of degrees ranging from 3 to 18, all GOF measures for the strength model (Equation 3) slightly improved with increasing degree, providing good fits for almost all degrees. However, the models with polynomial degrees ranging from 9 to 16 provided the most consistent estimates and *p* values. Thus, to avoid overfitting while still using a model with a relatively good fit as indicated by the GOF measures, we used the model with polynomial degree of 12 as a middle ground between 9 and 16. For the probability model, although all GOF measures slightly improved with increasing degree too, the differences were negligible. Thus, we used the same polynomial degree (12) for consistency. The estimates, standard errors, and *p* values for the polynomial parameters are presented in Table 2 (*p* values presented in this table and other tables are corrected for multiple comparison using an adaptive false discovery rate procedure detailed in Benjamini & Hochberg, 2000). The parameter estimates, standard errors, and *p* values (based on the residual approximation of the *F* test for a Wald statistic) associated with the important fixed-effect covariates are presented in Table 3. The estimates for other parameters (e.g., gender, age, etc.) are fully explained in the Supporting Information (Table S6). The estimates quantify the relationship between dynamic patterns of probability (presence/absence) and strength of (present) connections between nodes (brain regions), as dependent variables, and the previously mentioned sets of covariates, including (dynamic patterns of) endogenous network measures, fluid intelligence as our covariate of interest, and confounders. The estimates for confounding covariates, including sex, age, education, BMI, race, ethnicity, handedness, income, DSM4 alcohol abuse, DSM4 alcohol dependence, smoking status, spatial distance, and square of spatial distance between nodes are fully explained in the Supporting Information as pointed out above.

**Table 2.** Fixed-effect estimates, *SE*s, and *p* values for 12th degree orthonormal polynomial fit

Models	Parameters	Ortho Poly Degree	Estimate	*SE*	*P Value
Probability Model	γ_r,0_	Intercept	−0.13770	0.02022	<.0001
	γ_r,1_	1	0.00121	0.004361	0.7817
	γ_r,2_	2	−0.01694	0.004914	0.0021
	γ_r,3_	3	0.02394	0.004304	<.0001
	γ_r,4_	4	−0.00593	0.004641	0.3805
	γ_r,5_	5	−0.00508	0.004429	0.4498
	γ_r,6_	6	−0.01185	0.004382	0.0232
	γ_r,7_	7	0.00794	0.004019	0.1259
	γ_r,8_	8	0.00392	0.004106	0.4716
	γ_r,9_	9	−0.00433	0.004443	0.4716
	γ_r,10_	10	−0.01063	0.004236	0.0374
	γ_r,11_	11	0.00774	0.004203	0.1583
	γ_r,12_	12	−0.00481	0.004897	0.4716
Strength Model	γ_s,0_	Intercept	0.31190	0.00786	<.0001
	γ_s,1_	1	0.00148	0.00214	0.4906
	γ_s,2_	2	−0.01415	0.00221	<.0001
	γ_s,3_	3	0.01782	0.00203	<.0001
	γ_s,4_	4	−0.00110	0.00197	0.5785
	γ_s,5_	5	−0.00057	0.00214	0.7917
	γ_s,6_	6	−0.01012	0.00211	<.0001
	γ_s,7_	7	0.01077	0.00226	<.0001
	γ_s,8_	8	0.00388	0.00228	0.1106
	γ_s,9_	9	−0.00440	0.00204	0.0426
	γ_s,10_	10	−0.00726	0.00200	0.0005
	γ_s,11_	11	0.00134	0.00199	0.5007
	γ_s,12_	12	−0.00907	0.00257	0.0007

* Adjusted using the adaptive false discovery rate procedure described in Benjamini and Hochberg (2000).

**Table 3.** Parameter estimates, standard errors, and *p* values for dynamic networks

Probability Model Outputs				Strength Model Outputs			
Parameter	Estimate	*SE*	*P value	Parameter	Estimate	*SE*	*P value
β_r,0_	−0.1377	0.02022	<.0001	β_s,0_	0.31190	0.00786	<.0001
β_r,COI_	0.00319	0.00295	0.4716	β_s,COI_	−0.00086	0.00115	0.4540
β_r,C_	−7.22530	0.11490	<.0001	β_s,C_	3.07330	0.02008	<.0001
β_r,Eglob_	12.5799	0.43460	<.0001	β_s,Eglob_	3.86090	0.04105	<.0001
β_r,D_	−0.07686	0.00389	<.0001	β_s,D_	−0.07111	0.00672	<.0001
β_r,L_	0.04332	0.02006	0.0872	β_s,L_	−0.21300	0.00273	<.0001
β_r,Q_	2.10910	0.01471	<.0001	β_s,Q_	−1.35550	0.00241	<.0001
β_r,COI×C_	0.10290	0.11500	0.4836	β_s,COI×C_	−0.02418	0.02009	0.2478
β_r,COI×Eglob_	0.24720	0.43470	0.5696	β_s,COI×Eglob_	−0.00301	0.04106	0.9416
β_r,COI×D_	−0.00140	0.00389	0.7177	β_s,COI×D_	−0.00002	0.00672	0.9978
β_r,COI×L_	−0.01690	0.02006	0.4836	β_s,COI×L_	0.00127	0.00273	0.6423
β_r,COI×Q_	−0.02684	0.01514	0.1619	β_s,COI×Q_	**0.03078**	0.00248	**<.0001**

* Adjusted using the adaptive false discovery rate procedure described in Benjamini and Hochberg (2000). Bold values show fluid intelligence–related inferential results discussed here.

The estimates for interaction covariates shows if (and how) the relationship between dynamic patterns of probability/strength of connections and dynamic patterns of endogenous network measures are affected by fluid intelligence. Notable results are detailed in the following sections.

### Dynamic Network Analysis

As Table 3 presents, dynamic changes of clustering (functional segregation), global efficiency (functional integration), degree difference (functional resilience), and leverage centrality (information flow) all play important roles in explaining dynamic patterns of both connection probability and strength, but with leverage centrality having a marginal effect on dynamic patterns of connection probability.

Fluid intelligence, our covariate of interest (COI), is neither directly related to dynamic patterns of connection probability (presence/absence) nor connection strength as indicated by the *p* values associated with *β*_*r*,*COI*_ (*p* value = 0.4716) and *β*_*s*,*COI*_ (*p* value = 0.4540), respectively.

However, it has a significant effect on the relationship between dynamic changes of connection strength and dynamic changes of whole-brain modularity as indicated by the *p* value associated with *β*_*s*,*COI*×*Q*_ (*p* value < 0.0001), while having no effect on other relationships (using the gF as a median split binary [low/high] variable rather than a continuous one, provided the same results as shown in Supporting Information Appendix S5). Dynamic changes of whole-brain modularity and connection strength are negatively associated with each other (*β*_*s*,*Q*_), which implies the dominance of between-community (rather than within-community) connections in driving the dynamic changes of whole-brain modularity. Fluid intelligence interacts with this relationship—as intelligence increases, dynamic changes of modularity are less driven by dynamic changes in between-community (and more by within-community) connectivity as indicated by the positive and significant estimate for *β*_*s*,*COI*×*Q*_. (However, the dynamics of between-community connections are still the dominant factor in driving the dynamic changes of whole-brain modularity.)

**Figure 3.**
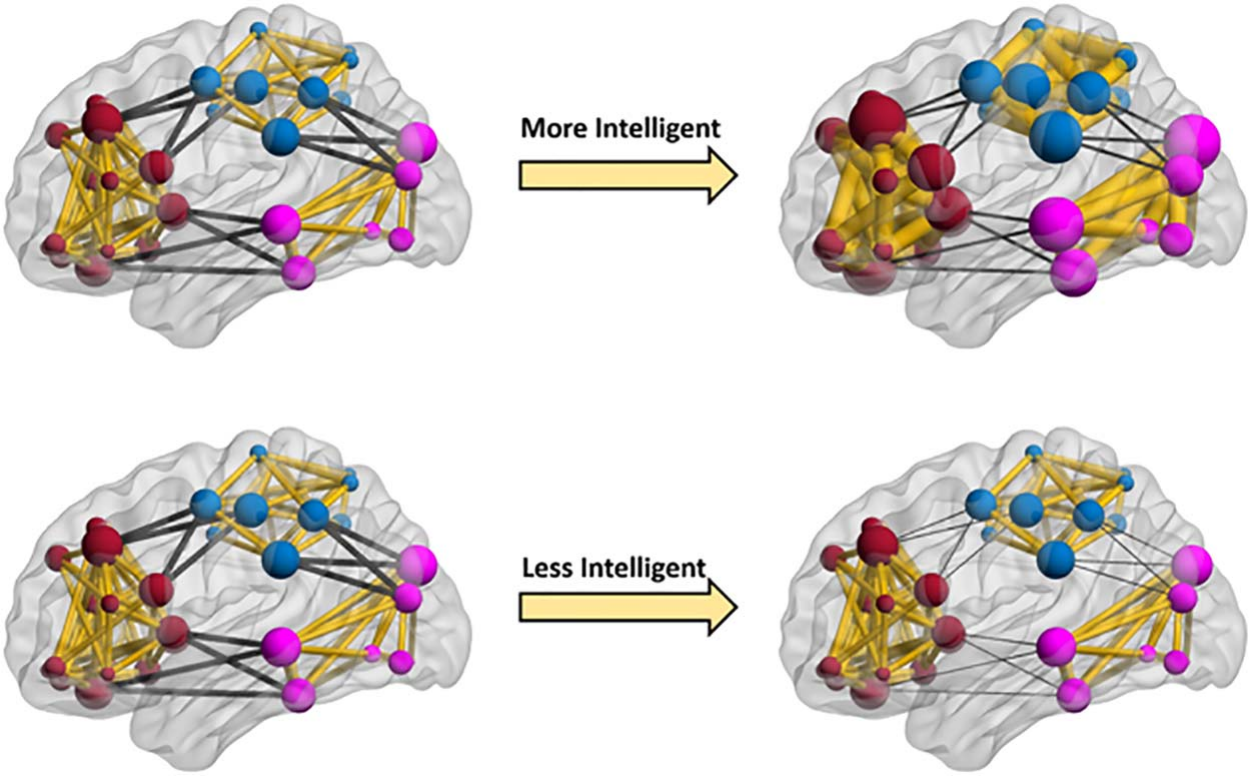
Cartoon depiction of fluid intelligence effects on dynamic brain networks. The nodes represent brain regions, and edges represent dynamic functional connections. To illustrate the effects of fluid intelligence on dynamic changes of modularity as interpreted from Table 3, three artificial communities marked with separate colors (dark red, light blue, and purple) are shown in this figure. The within- and between-community connections are shown with the yellow and black colors, respectively. As this figure illustrates, dynamic changes of modularity are predominantly determined by between-community connections for any level of intelligence (here two levels are shown: low and high). However, when comparing the more and less intelligent participants (networks on the right), in more intelligent participants, dynamic changes of modularity are less determined by between-community connections (thicker dark edges in top right), and dynamic changes of within-community connections also play more important roles in changing the modularity (thicker yellow edges in top right network). We should note that fluid intelligence was modeled as a continuous variable, but for illustrative purposes we show higher and lower intelligence levels. We have made a movie that better illustrates how dynamic patterns of whole-brain modularity are affected across a range of fluid intelligence values (Supporting Information Movie S1).

Our results might imply that brain networks in people with higher fluid intelligence are more flexible with respect to changes in their modularity at rest. These changes are associated with both stronger within-community connections (more specialized neural communities) and weaker between-community connections (more segregated neural communities). This is illustrated in Figure 3 and Supporting Information Movie S1. We provide more detail on possible interpretations and implications of this result in the Discussion. The results for using windows with 50% overlap between consecutive networks were similar, but with gF modifying the relationship between dynamic patterns of whole-brain modularity and connection probability as well. For more detail and a brief interpretation see Supporting Information Appendix S2.

### Dynamic Network Simulation

Using the estimated parameters from fitted models in Equations 2 and 3, we simulated 5,000 dynamic networks: 10 realizations for each one of 10 (randomly selected) dynamic networks from 50 (randomly selected) individuals. We then calculated descriptive graph measures including: C, Eglob, and k. Table 4 presents the average values across all dynamic networks and all regions for both observed and simulated networks.

**Table 4.** Weighted network measures of observed and simulated dynamic networks

Metric	Observed (*N* = 50 × 10)		Simulated (*N* = 50 × 10 × 10)	
	Mean	*SD*	Mean	*SD*
Clustering coefficient (C)	0.1778	0.0217	0.1463	0.0296
Global efficiency (Eglob)	0.2948	0.0096	0.3263	0.0517
Degree (K)	39.229	2.8391	40.847	7.3629

**Figure 4.**
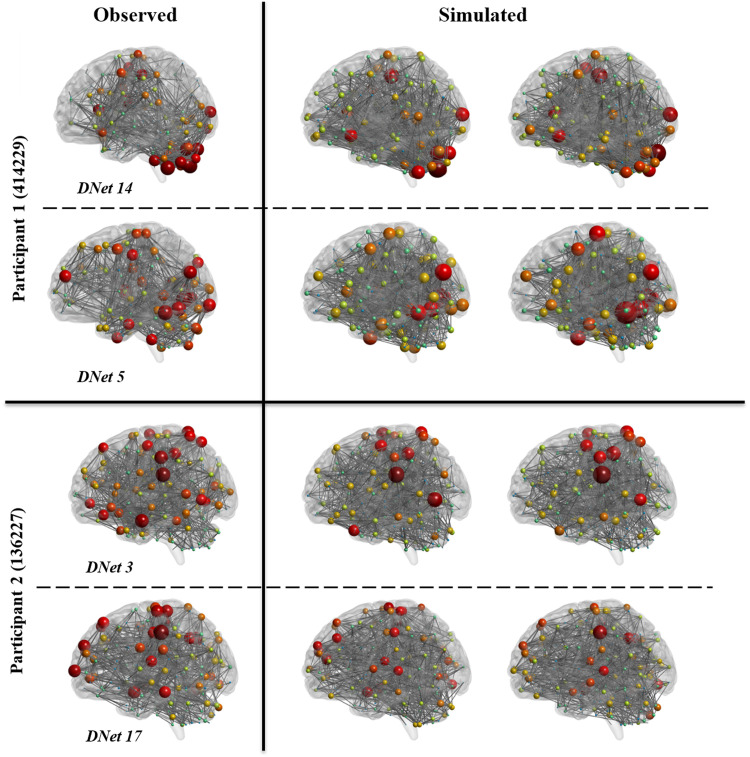
Observed and simulated dynamic networks for two randomly selected participants. For each participant, two randomly selected dynamic networks (DNet #) are shown on the left, and for each dynamic network, two randomly selected simulated networks (from the 10 simulation realizations) are shown on the right. We have shown the HCP individual IDs on the left. All networks are thresholded to maintain the top 5% of strongest connections. The size and color of each node represent the degree of that node.

As evidenced by Table 4, average network measures are very close between simulated and observed networks, indicating the ability of this model to simulate representative group-level dynamic networks. However, to further illustrate that simulated networks represent observed dynamic networks at multiple resolutions beyond average values, that is, at the individual and nodal level, in Figure 4, we have shown two realizations of two dynamic networks for two participants (all chosen randomly from the 5,000 simulated networks). Networks in this figure represent the top 5% of strongest connections. As this figure shows, even after thresholding the networks, the simulated networks well represent the observed networks.

## DISCUSSION

As the interest in dynamic brain networks continues to grow, new methods are needed to enable gleaning neurobiological insight into this complex and big data. Development of multivariate statistical methods, particularly model-based ones, which allow quantifying relationships between phenotypic traits and dynamic patterns of brain connectivity and topology, as well as drawing inference from such relationships, is among the urgent needs. Development of such methods even for static networks has remained a challenge given the size, complexity, and multiscale dependence inherent in brain network data. However several model-based methods (Shehzad et al., 2014; Simpson, Hayasaka, & Laurienti, 2011; Simpson & Laurienti, 2015) and various data-driven multivariate methods (Allen et al., 2011; Beckmann & Smith, 2004; Calhoun, Adali, Pearlson, & Pekar, 2001; Smith, Hyvarinen, Varoquaux, Miller, & Beckmann, 2014) have been introduced and extensively used for static networks. Dynamic changes in the systematic organization of our brain networks confer much of our brains’ functions abilities due to the fact that our brain is a complex multiscale dynamic system with known and unknown compensatory mechanisms at multiple scales. Thus, methods that allow analyzing the brain within a multivariate framework can provide much deeper insights into dynamic patterns of brain networks in health and disease. In addition, multivariate model-based tools enable aligning neuroscientific hypotheses with the analytic approach, which is ideal for dynamic brain network analysis (Preti, Bolton, & Van De Ville, 2017). Nevertheless, no model-based multivariate method has been introduced for dynamic network analyses to our knowledge.

Here we provided a model-based multivariate method to relate phenotypic traits to dynamic patterns of brain connectivity and topology. We developed this model by advancing a two-part mixed-effects regression framework for static brain networks (Simpson & Laurienti, 2015). Our proposed model allows accounting for the connectivity/network dynamics when assessing group differences and phenotype-health outcome relationships, to avoid confounding and drawing erroneous conclusions. The incorporation of endogenous network measures such as clustering coefficient and global efficiency, as independent variables, allows simultaneous analyses of connectivity and topology dynamics. There is a long history of modeling a network as a function of endogenous network metrics to identify how nodal properties are related to the probability (and strength) of connections (O’Malley, 2013; Robins, Pattison, Kalish, & Lusher, 2007; Simpson et al., 2011, 2012). Part of the motivation for our modeling framework was the desire to port this approach into the time-varying multiple-network context and blend it with the more standard exogenous covariate approach to create a hybrid method that allows examining and accounting for both an individual’s endogenous network structure and exogenous phenotypic characteristics in a manner suitable for dynamic brain network analyses. The topological network covariates allow examining how nodal properties influence the connection between two brain areas. Having both the endogenous and exogenous covariates in the model allows us to assess how these topology-connection relationships vary by individual and group characteristics. Additionally, it allows simulating more realistic dynamic networks by incorporating the dynamics of an array of explanatory network measures and their interplay with desired covariates.

Most current methods used to assess dynamic brain networks reduce this data into dynamic patterns of individual brain connections (Schmlazle et al., 2017; Simony et al., 2016; Tewarie et al., 2019) or commonly used topological summary variables, such as node degree or modularity (Jones et al., 2012; Kabbara et al., 2019), rather than analyzing the systemic dynamics of the brain networks. Such methods not only fail to model the brain as a multiscale dynamic system (Lurie et al., 2020), but often entail matching study populations to perform group comparisons, which is a daunting task for most neuroimaging studies. Our model provides a framework to assess the systemic dynamics of brain networks and thus to account for complex dynamics of the brain via the simultaneous modeling of brain connectivity and topological network variables. The multivariate nature of this framework reduces demands for matching study populations as any number of confounding effects can be incorporated as covariates, and the effects of multiple covariates of interest can be studied in a single model. Another important utility of this model is its ability to simulate dynamic brain networks, which is critical for a better understanding of brain function in health and disease (Tikidji-Hamburyan, Narayana, Bozkus, & El-Ghazawi, 2017). To our knowledge, our framework is the first model that allows simulating dynamic brain networks from system-level properties of the brain, and with respect to desired covariates. An important utility of the simulation capability of this model is to generate representative group-level dynamic networks. The need for reliably generating representative group-level networks has been well documented (Jirsa, Sporns, Breakspear, Deco, & McIntosh, 2010; Meunier, Achard, Morcom, & Bullmore, 2009; Valencia et al., 2009; Zuo et al., 2012). Additionally, the simulation capability also provides a scientifically appropriate way to assess GOF (as shown in the Dynamic Network Simulation section) and simulate individual-level networks.

It is important to note that our framework itself, which aims to account for continuous time-varying network changes when relating covariates to topology, is not designed to identify latent states like hidden Markov models are, for example. But adding a latent state analysis to the preprocessing steps prior to implementing our model would provide a complementary and insightful extension to our overall approach. Our method will work for many approaches used to generate the networks, allowing for great flexibility in the network generation method that one chooses to use. A standard approach for assessing the performance of regression-based methods is through examining the quality of model fit. Since our method is also a regression-based framework, we used different GOF measures to examine the performance of our method. More importantly, our simulation analyses allowed for a more profound assessment of the performance of our method as the most appropriate method to assess the GOF of statistical methods in the network context is through simulation analyses (Hunter et al., 2008). In addition, with respect to identifying the association between phenotypic traits and dynamic brain networks, our method uses a fundamentally different approach, and provides different (complementary) insight, than current data-driven methods, and thus a comparison between our model and current data-driven models would not be appropriate.

We demonstrated the utility of our model in identifying the relationship between fluid intelligence and dynamic patterns of brain connectivity and topological network variables by using the rich data set provided by the HCP study (Van Essen et al., 2013). Our model allowed accounting for various sources of potential confounding effects, such as sex, education, age, and alcohol abuse, among others. Our results indicated that dynamic patterns of whole-brain modularity and connection strength are significantly affected by fluid intelligence. More specifically, our results showed that for any level of fluid intelligence, dynamic patterns of modularity are predominantly associated with between-community, rather than within-community, connections. However, fluid intelligence modulates this trend such that, across an entire spectrum of fluid intelligence, dynamics of whole-brain modularity play a less important role in driving changes in between-community connections for higher fluid intelligence values (with dynamics of within-community connections probably being affected more). While the ultimate neurobiological interpretations of such effects is speculative at this point, our results may suggest that at lower levels of intelligence, distinct network modules necessary for cognition (such as the module comprised of areas forming the central executive attention network, or CEN) are formed primarily by segregating information. Thus, distinct subnetworks are formed by decreasing connectivity to other subnetworks, which could result in relatively poor distribution of information between subnetworks. As intelligence increases, the formation of distinct modules is driven more by strengthening connections within the module and less by segregating modules. Increased intramodular connectivity could enhance processing within subnetworks like the CEN while maintaining communication between subnetworks for optimal information distribution. Other studies have reported associations between brain modularity and intelligence (Chaddock-Heyman et al., 2020), as well as significant correlations between creativity and learning and dynamic patterns of brain modularity (Bassett et al., 2011; Kenett, Betzel, & Beaty, 2020).

In the absence of a “gold standard” in sliding window approach, the optimal choice for window type, window length, and step size is challenging. We used a modulated rectangular window due to its superior performance in examining dynamic brain networks when compared to other conventional window types (Mokhtari, Akhlaghi et al., 2019). We used 120 volumes for our window length for multiple reasons, including (1) to provide more stabilized correlation values while not losing the variability of the brain dynamics, (2) due to its wider use which makes comparing and contrasting our method with currently used methods easier, and (3) model fit and convergence considerations of our proposed method. It is also important to note that no commonly used window length can accurately identify different states of correlation (Shakil, Lee, & Keilholz, 2016), and that the sliding window correlation approach is only used to demonstrate the utility of our method rather than to provide comprehensive analyses of fluid intelligence-dynamic brain network associations. Also, typical shift sizes used in the literature range from 1 TR to 50% of the window length (Chang et al., 2013; Kucyi & Davis, 2014; Shakil, Magnuson, Keilholz, & Lee, 2014), with the 1 TR being the most commonly used shift size (Shakil et al., 2016). However, as our proposed method subsequently uses the dynamic networks in a regression framework, we used a shift size of 120 volumes, equal to the window length, to create nonoverlapping windows and thus further reduce autocorrelation. Additionally, using nonoverlapping windows allowed using a smaller, but sufficient number of dynamic networks for each participant and thus helped avoiding possible convergence issues. Nevertheless, for windows with 50% overlap between consecutive networks, our modeling framework yielded similar outcomes.

Incorporating all brain connections and modeling the dependence among multiple variables across time is computationally time intensive and can lead to convergence issues for datasets with large numbers of participants and regions of interest. This prevented us from using more complex variance-covariance structures for the random effects that could, in turn, yield even more accurate estimates and better simulations of dynamic brain networks. We plan to develop complementary data reduction methods to address this in future work. Moreover, there is no agreement on a well-accepted atlas for functional connectivity studies. While many studies have used clustering-based methods or canonical correlation analysis to define nodes for their own data (Allen et al., 2014), other studies have used publicly available parcellation schemes generated from rich datasets with higher reproducibility across individuals (Glasser et al., 2016). Even newer studies indicate that using an individual atlas rather than a group-level parcellation may provide more reliable results (Salehi et al., 2020). However, as with the parameters used for generating the dynamic networks (sliding window type, length, etc.), our method is independent of the parcellation scheme used for defining the brain regions. The sensitivity to the method used to generate dynamic networks as well as the parcellation scheme (Bahramf & Hossein-Zadeh, 2014; Glasser et al., 2016; Power et al., 2011) will be assessed in future studies as for each parameter, multiple models should be run to find the best orthonormal polynomial degree for that particular parameter, and thus, a composite grid search over all combinations of such parameters and polynomial degrees will be required, which is beyond the scope of this paper. The proposed mixed-effects framework can be used for predicting dynamic networks based on participant characteristics as well. However, we didn’t demonstrate this capability here. These capabilities will be demonstrated in future work, given that they require extensive analytical assessment and exposition, which lie beyond the scope of this paper. We have also made our proposed framework accessible to neuroimaging researchers by incorporating new graphical user interfaces into WFU_MMNET (https://www.nitrc.org/projects/wfu_mmnet), the software developed for the application of the original static model (Bahrami et al., 2019b).

This study is not without limitations. Incorporating all brain connections as well modeling the dependence structure between multiple variables across individuals’ brain connections and network metrics (e.g., clustering coefficient, global efficiency) across time is computationally intensive and can lead to convergence issues (particularly for the probability model; Equation 2) for datasets with large numbers of participants and network nodes. This precludes using our method for studies analyzing voxel level dynamic networks and limits its utility for studies with very large numbers of subjects or very spatially resolved (>1,000 nodes) brain networks. We have developed a clustering-based data reduction method to resolve this issue for the original model for static networks and are currently working on extending it to be applicable for dynamic brain networks as well. This will greatly aid in resolving the convergence issue. Also, parcellation schemes generated from a clustering or canonical correlation analysis approach with a fewer number of regions of interest are also appealing alternatives to be used within our framework. Interpreting the results produced by our method may not always be straightforward, as both connectivity and network topology dynamics should be considered simultaneously. This can complicate interpretation of the results for continuous variables and when particular network metrics with both local and global implications are examined, such as clustering coefficient. Another limitation of our method is that its only applicable for whole-brain networks at present, and not for analyzing dynamics of local brain networks or subnetworks (e.g., the default mode network) unless they are extracted from the whole-brain network. However, we are working on devising a procedure to allow for analyzing dynamics of local brain networks within the context of their whole networks as was done for the original framework for static networks in Bahrami et al. (2019a). Finally, future studies should conduct a thorough sensitivity analysis of our framework with respect to the parameters used in generating the dynamics networks and the parcellation scheme used.

## SUPPORTING INFORMATION

Supporting information for this article is available at https://doi.org/10.1162/netn_a_00238.

## AUTHOR CONTRIBUTIONS

Mohsen Bahrami: Data curation; Formal analysis; Methodology; Validation; Visualization; Writing – original draft; Writing – review & editing. Paul J. Laurienti: Conceptualization; Funding acquisition; Validation; Writing – review & editing. Heather M. Shappell: Conceptualization; Writing – review & editing. Dale Dagenbach: Conceptualization; Writing – review & editing. Sean L. Simpson: Conceptualization; Funding acquisition; Methodology; Supervision; Validation; Writing – original draft; Writing – review & editing.

## FUNDING INFORMATION

Sean L. Simpson, National Institute of Biomedical Imaging and Bioengineering (https://grantome.com/grant/NIH/R01-EB024559-04), Award ID: R01EB024559.

## TECHNICAL TERMS

Dynamic brain networks: Large-scale brain networks that vary over short periods of time on the order of seconds.
System-level properties: Properties of the brain as a system comprising multiple interacting regions.
Mixed-effects (or mixed) models: Statistical regression models that include both fixed (i.e., population-level) and random (i.e., individual-level) parameters.
Fixed effects: Variables in the mixed models whose effects are constant across all individuals.
Random effects: Variables in the mixed models whose effects change across individuals.
Orthonormal polynomials: A family of orthogonal polynomials where the inner product of the same polynomials is one.
Bernoulli distribution: Discrete probability distribution for a random variable with binary (i.e., 0 or 1) outcome.
