# Supplementary material for: A mixed-modeling framework for whole-brain dynamic network analysis
Source: Netw Neurosci. 2022 Jun 1;6(2):591–613. doi: 10.1162/netn_a_00238 (PMC9208000; doi:10.1162/netn_a_00238)
Supplement: Supplementary file 1 [file netn-06-591-s001.pdf]

Appendix S1. HCP subject identification numbers

HCP identification number for the 200 subjects used in this study are provided in Table S1 below.

Table S1. HCP subject identification numbers

100206	101410	104012	107321	107422	107725	110411	111413
111514	114217	114823	114924	115017	117021	117122	118225
118730	118831	119126	121618	123420	123925	124826	126426
127630	127933	128935	129028	129634	130316	131924	132017
133827	134021	135124	135225	135932	136227	138332	139637
140319	142424	143426	144731	145834	146432	146533	147030
148335	148941	149337	150928	151223	151425	152831	153833
154936	155938	158136	159138	160729	161832	165638	169141
169747	169949	170934	171330	172130	172433	172534	173132
173334	173435	175742	176037	177645	178142	178243	178950
179346	180937	182032	182436	183034	185038	186141	186444
188145	188751	190031	192035	192136	193239	195041	196750
197348	199150	199655	200109	201414	201818	204319	206222
206323	207123	207426	208226	209935	210112	213522	214019
214423	214524	219231	220721	223929	227432	237334	239136
245333	263436	268749	270332	274542	280739	280941	299154
317332	325129	334635	342129	346945	349244	358144	376247
377451	378756	380036	389357	391748	397154	406836	412528
414229	419239	424939	432332	436845	445543	448347	469961
485757	500222	512835	513130	517239	518746	519647	522434
523032	529549	540436	541640	553344	555651	559053	561444
571144	578158	579665	583858	597869	613538	618952	623137
623844	647858	654552	656253	656657	672756	685058	688569
689470	690152	695768	701535	709551	713239	729557	732243
744553	757764	760551	766563	769064	770352	771354	802844

Appendix S2. Analysis for networks with 50% overlap

Results for using windows with 50% overlap between consecutive networks are shown in Table S2 below. Given that dynamic patterns of whole-brain modularity and connection probability are positively associated with each other ($\beta_{r,Q}$), and the estimate for $\beta_{r,COI\times Q}$ shows a negative modification effect from gF, this result implies that in more intelligent people, dynamic patterns of whole-brain modularity are more driven by a reduction in the number of between community connections when compared to less intelligent people. The results for the strength model are the same as those for windows with no overlap between consecutive networks and thus can be interpreted in the same way. We have shown the results for orthonormal polynomial degree of 12 for consistency (however, we ran this analysis for polynomial degrees ranging from 3-25, and models with polynomial degrees of 12 and higher provided the most consistent results with all GOF measures improving slightly).

Table S2. Parameter estimates, standard errors, and p-values for windows with 50% overlap

**Probability Model Outputs**				**Strength Model Outputs**			
Parameter	Estimate	SE	*p-value	Parameter	Estimate	SE	*p-value
$\beta_{r,0}$	-0.13550	0.01800	<.0001	$\beta_{s,0}$	0.30880	0.06809	<.0001
$\beta_{r,COI}$	0.00237	0.00263	0.3676	$\beta_{s,COI}$	-0.00029	0.00993	0.9771
$\beta_{r,C}$	-6.92290	0.10510	<.0001	$\beta_{s,C}$	2.97360	0.02049	<.0001
$\beta_{r,Eglob}$	12.0428	0.38040	<.0001	$\beta_{s,Eglob}$	2.99390	0.03514	<.0001
$\beta_{r,D}$	-0.07514	0.00373	<.0001	$\beta_{s,D}$	-0.06858	0.00024	<.0001
$\beta_{r,L}$	0.04535	0.01487	0.0048	$\beta_{s,L}$	-0.15030	0.00167	<.0001
$\beta_{r,Q}$	2.00170	0.00830	<.0001	$\beta_{s,Q}$	-1.13950	0.00134	<.0001
$\beta_{r,dist}$	-0.15500	0.00312	<.0001	$\beta_{s,dist}$	-0.02723	0.00039	<.0001
$\beta_{r,dist2}$	0.07730	0.00154	<.0001	$\beta_{s,dist2}$	0.01625	0.00021	<.0001
$\beta_{r,Gender}$	0.00665	0.00492	0.2077	$\beta_{s,Gender}$	-0.00464	0.01862	0.8034
$\beta_{r,Age}$	0.00567	0.00267	0.0477	$\beta_{s,Age}$	0.00142	0.01009	0.8878
$\beta_{r,EduLev1}$	0.01216	0.00931	0.2154	$\beta_{s,EduLev1}$	0.00301	0.03522	0.9319
$\beta_{r,EduLev2}$	0.00961	0.00666	0.1827	$\beta_{s,EduLev2}$	0.00139	0.02520	0.9559
$\beta_{r,BMI}$	0.00111	0.00247	0.6516	$\beta_{s,BMI}$	0.00021	0.00934	0.9822
$\beta_{r,RaceAm}$	-0.00603	0.03418	0.8600	$\beta_{s,RaceAm}$	-0.01472	0.12930	0.9094
$\beta_{r,RaceAsian}$	-0.00122	0.00895	0.8912	$\beta_{s,RaceAsian}$	-0.00166	0.03389	0.9610
$\beta_{r,RaceBlack}$	0.00357	0.00794	0.6529	$\beta_{s,RaceBlack}$	0.00658	0.03002	0.8265
$\beta_{r,RacedMore}$	-0.00614	0.01900	0.7467	$\beta_{s,RacedMore}$	-0.00966	0.07187	0.8931
$\beta_{r,RaceUknown}$	-0.00115	0.01817	0.9494	$\beta_{s,RaceUknown}$	-0.00328	0.06877	0.9620
$\beta_{r,EthnHisp}$	0.02042	0.01794	0.2649	$\beta_{s,EthnHisp}$	0.00519	0.06787	0.9390
$\beta_{r,EthnNonHisp}$	0.01478	0.01713	0.3881	$\beta_{s,EthnNonHisp}$	0.00455	0.06480	0.9440
$\beta_{r,Handedness}$	0.00064	0.00244	0.7917	$\beta_{s,Handedness}$	-0.00062	0.00923	0.9463
$\beta_{r,Income}$	0.00148	0.00124	0.2492	$\beta_{s,Income}$	0.00095	0.00469	0.8398

$\beta_{r,AlcAbuse}$	0.00152	0.00625	0.8077	$\beta_{s,AlcAbuse}$	-0.00005	0.02364	0.9984
$\beta_{r,AlcDep}$	-0.00047	0.00897	0.9582	$\beta_{s,AlcDep}$	-0.00278	0.03395	0.9347
$\beta_{r,SmokStatus}$	0.00382	0.00683	0.5757	$\beta_{s,SmokStatus}$	0.00065	0.02583	0.9800
$\beta_{r,COI\times C}$	0.10650	0.10520	0.3111	$\beta_{s,COI\times C}$	-0.01807	0.02049	0.4270
$\beta_{r,COI\times Eglob}$	0.35420	0.38050	0.3519	$\beta_{s,COI\times Eglob}$	0.03335	0.03515	0.4051
$\beta_{r,COI\times D}$	-0.00292	0.00373	0.4340	$\beta_{s,COI\times D}$	-0.00020	0.00024	0.4312
$\beta_{r,COI\times L}$	-0.02583	0.01487	0.1113	$\beta_{s,COI\times L}$	-0.00159	0.00167	0.4051
$\beta_{r,COI\times Q}$	**-0.09576**	0.00858	**<.0001**	$\beta_{s,COI\times Q}$	**0.00424**	0.00138	**0.0031**

* Adjusted using the adaptive FDR procedure described in (Benjamini, Hochberg et al. 2000).

Appendix S3. Static network analysis

In this analysis, we examined the effects of fluid intelligence (gF) on static brain networks for comparison. We used the original mixed models introduced in (Simpson and Laurienti 2015) with the exact same fixed- and random-effects, and the same random-effects variance covariance structure as those used for dynamic networks. The results (Table S3) clearly illustrate that different conclusions would be drawn from employing this modeling approach, particularly when comparing the effects of gF on brain modularity and connectivity. While dynamic patterns of whole-brain modularity are modified by fluid intelligence, the static network analyses indicate no such effect.

Table S3. Parameter estimates, standard errors, and p-values for analyzing static networks

**Probability Model Outputs**				**Strength Model Outputs**			
Parameter	Estimate	SE	*p-value	Parameter	Estimate	SE	*p-value
$\beta_{r,0}$	-0.2584	0.0809	0.0014	$\beta_{s,0}$	0.1022	0.0289	0.0004
$\beta_{r,COI}$	-0.2073	0.0110	0.2793	$\beta_{s,COI}$	-0.0089	0.0043	0.0369
$\beta_{r,C}$	-2952.4	46.959	<0.0001	$\beta_{s,C}$	0.0777	0.0008	<0.0001
$\beta_{r,Eglob}$	-0.0403	0.0079	<0.0001	$\beta_{s,Eglob}$	0.0700	0.0009	<0.0001
$\beta_{r,D}$	0.0176	0.0042	<0.0001	$\beta_{s,D}$	-0.0433	0.0005	<0.0001
$\beta_{r,L}$	0.1714	0.0092	<0.0001	$\beta_{s,L}$	-0.0419	0.0009	<0.0001
$\beta_{r,Q}$	0.0918	0.0110	<0.0001	$\beta_{s,Q}$	-0.0177	0.0039	<0.0001
$\beta_{r,dist}$	-0.2993	0.0050	<0.0001	$\beta_{s,dist}$	-0.0398	0.0005	<0.0001
$\beta_{r,dist2}$	0.1510	0.0024	<0.0001	$\beta_{s,dist2}$	0.0211	0.0003	<0.0001
$\beta_{r,Gender}$	0.0022	0.0220	0.9197	$B_{s,Gender}$	0.0121	0.0079	0.1263
$\beta_{r,Age}$	0.0025	0.0119	0.8350	$\beta_{s,Age}$	0.0043	0.0043	0.3092
$\beta_{r,EduLev1}$	0.0524	0.0415	0.2070	$\beta_{s,EduLev1}$	0.0018	0.0149	0.9062
$\beta_{r,EduLev2}$	0.0164	0.0298	0.5822	$\beta_{s,EduLev2}$	0.0101	0.0107	0.3471
$\beta_{r,BMI}$	0.0232	0.0114	0.0411	$\beta_{s,BMI}$	-0.0086	0.0041	0.0348
$\beta_{r,RaceAm}$	0.0967	0.1523	0.5253	$\beta_{s,RaceAm}$	-0.0816	0.0549	0.1374
$\beta_{r,RaceAsian}$	-0.0038	0.0400	0.9250	$\beta_{s,RaceAsian}$	-0.0107	0.0144	0.4570
$\beta_{r,RaceBlack}$	-0.0317	0.0359	0.3777	$\beta_{s,RaceBlack}$	0.0157	0.0129	0.2234
$\beta_{r,RacedMore}$	0.1128	0.0842	0.1803	$\beta_{s,RacedMore}$	-0.0109	0.0304	0.7209
$\beta_{r,RaceUknown}$	0.0248	0.0817	0.7618	$\beta_{s,RaceUknown}$	0.0043	0.0295	0.8843
$\beta_{r,EthnHisp}$	-0.0965	0.0808	0.2320	$\beta_{s,EthnHisp}$	0.0464	0.0289	0.1083
$\beta_{r,EthnNonHisp}$	-0.0580	0.0769	0.4507	$\beta_{s,EthnNonHisp}$	0.0393	0.0275	0.1536
$\beta_{r,Handedness}$	0.0081	0.0109	0.4531	$\beta_{s,Handedness}$	-0.0041	0.0039	0.2915
$\beta_{r,Income}$	-0.0098	0.0122	0.4266	$\beta_{s,Income}$	0.0042	0.0044	0.3429
$\beta_{r,AlcAbuse}$	0.0311	0.0279	0.2637	$\beta_{s,AlcAbuse}$	0.0063	0.0100	0.5313
$\beta_{r,AlcDep}$	-0.0162	0.0402	0.6870	$\beta_{s,AlcDep}$	-0.0082	0.0145	0.5726
$\beta_{r,SmokStatus}$	-0.0628	0.0307	0.0409	$\beta_{s,SmokStatus}$	0.0034	0.0110	0.7616
$\beta_{r,COI\times C}$	-0.0069	0.0111	0.5333	$\beta_{s,COI\times C}$	0.0009	0.0008	0.2866
$\beta_{r,COI\times Eglob}$	0.0026	0.0079	0.7475	$\beta_{s,COI\times Eglob}$	-0.0009	0.0009	0.2927
$\beta_{r,COI\times D}$	-0.0016	0.0042	0.7117	$\beta_{s,COI\times D}$	-0.0005	0.0005	0.2367
$\beta_{r,COI\times L}$	0.0033	0.0092	0.7226	$\beta_{s,COI\times L}$	-0.0012	0.0009	0.1988
$\beta_{r,COI\times Q}$	0.0055	0.0117	0.6401	$\beta_{s,COI\times Q}$	-0.0024	0.0042	0.5592

* Adjusted using the adaptive FDR procedure described in (Benjamini, Hochberg et al. 2000).

Appendix S4. Orthonormal polynomials

The orthonormal term in equation 2 can be further expanded as below:

$$logit\left(p_{ijkt}(\boldsymbol{\beta}_r; \boldsymbol{b}_{ri}; \boldsymbol{\gamma}_r; \boldsymbol{d}_{ri})\right) = \cdots + \sum_{o=1}^{n} \gamma_{ro} s_{(o)}(X_t) + \cdots$$

$$\sum_{o=1}^{n} \gamma_{ro} s_{(o)}(X_t) = \left([\,\gamma_{r1} \dots\ \gamma_{rn}] \begin{bmatrix} s_{(1)} \\ \vdots \\ s_{(n)} \end{bmatrix} \right) X_t = \left([\,\gamma_{r1} \dots\ \gamma_{rn}] \begin{bmatrix} s_{1,t1} & \cdots & s_{1,T} \\ \vdots & \ddots & \vdots \\ s_{n,t1} & \cdots & s_{n,T} \end{bmatrix} \right) X_t$$

$$= \begin{cases} \gamma_{r1} s_{1,ti} + \gamma_{r2} s_{2,ti} + \cdots \gamma_{rn} s_{n,ti}\,, & for\ t = ti \\ zero \qquad\qquad\qquad\qquad\qquad\ , & otherwise \end{cases}$$

For example, for dynamic networks with 9 sliding windows, we will have the following matrix representing generated values from a set of orthonormal polynomials with maximum degree of 8 (9-1):

1	-0.5164	0.5318	-0.4449	0.3129	-0.1849	0.0899	-0.0341	0.0088
2	-0.3873	0.133	0.2225	-0.4693	0.5085	-0.382	0.2048	-0.0705
3	-0.2582	-0.1519	0.4132	-0.2458	-0.1849	0.4944	-0.478	0.2468
4	-0.1291	-0.3229	0.286	0.2011	-0.416	0.0225	0.478	-0.4936
5	0	-0.3799	0	0.4023	0	-0.4495	0	0.617
6	0.1291	-0.3229	-0.286	0.2011	0.416	0.0225	-0.478	-0.4936
7	0.2582	-0.1519	-0.4132	-0.2458	0.1849	0.4944	0.478	0.2468
8	0.3873	0.133	-0.2225	-0.4693	-0.5085	-0.382	-0.2048	-0.0705

The random effect term for orthonormal polynomials can be expanded in the same way.

Appendix S5. Modeling gF as binary variable

Table S4. Parameter estimates, standard errors, and p-values for using gF as a binary variable

Probability Model Outputs				Strength Model Outputs			
Parameter	Estimate	SE	*p-value	Parameter	Estimate	SE	*p-value
$\beta_{r,0}$	-0.13270	0.02085	<.0001	$\beta_{s,0}$	0.31330	0.00811	<.0001
$\beta_{r,COI}$	-0.00203	0.00574	0.7250	$\beta_{s,COI}$	-0.00274	0.00223	0.2428
$\beta_{r,C}$	-7.24620	0.15980	<.0001	$\beta_{s,C}$	3.09400	0.02737	<.0001
$\beta_{r,Eglob}$	12.5252	0.60340	<.0001	$\beta_{s,Eglob}$	3.86350	0.05602	<.0001
$\beta_{r,D}$	-0.07702	0.00539	<.0001	$\beta_{s,D}$	-0.07093	0.00920	<.0001
$\beta_{r,L}$	0.04280	0.02787	0.2492	$\beta_{s,L}$	-0.21440	0.00388	<.0001
$\beta_{r,Q}$	2.14100	0.02107	<.0001	$\beta_{s,Q}$	-1.41130	0.00346	<.0001
$\beta_{r,dist}$	-0.15460	0.00313	<.0001	$\beta_{s,dist}$	-0.02733	0.00038	<.0001
$\beta_{r,dist2}$	0.07723	0.00156	<.0001	$\beta_{s,dist2}$	0.01615	0.00021	<.0001
$\beta_{r,Gender}$	0.00615	0.00557	0.4548	$\beta_{s,Gender}$	-0.00789	0.00217	0.0005
$\beta_{r,Age}$	0.00508	0.00300	0.1940	$\beta_{s,Age}$	0.00149	0.00117	0.2365
$\beta_{r,EduLev1}$	0.00290	0.01036	0.7792	$\beta_{s,EduLev1}$	0.00246	0.00403	0.5417
$\beta_{r,EduLev2}$	0.00591	0.00749	0.5507	$\beta_{s,EduLev2}$	0.00213	0.00291	0.4655
$\beta_{r,BMI}$	0.00156	0.00278	0.6143	$\beta_{s,BMI}$	0.00076	0.00108	0.4795
$\beta_{r,RaceAm}$	0.00478	0.03854	0.9014	$\beta_{s,RaceAm}$	-0.01821	0.01498	0.2428
$\beta_{r,RaceAsian}$	0.00569	0.01018	0.6143	$\beta_{s,RaceAsian}$	-0.00297	0.00396	0.4535
$\beta_{r,RaceBlack}$	0.00017	0.00889	0.9845	$\beta_{s,RaceBlack}$	0.00675	0.00346	0.0663
$\beta_{r,RacedMore}$	-0.00610	0.02139	0.7755	$\beta_{s,RacedMore}$	-0.00968	0.00832	0.2542
$\beta_{r,RaceUknown}$	-0.00242	0.02056	0.9063	$\beta_{s,RaceUknown}$	-0.00785	0.00800	0.3266
$\beta_{r,EthnHisp}$	0.01767	0.02022	0.5320	$\beta_{s,EthnHisp}$	0.00620	0.00786	0.4303
$\beta_{r,EthnNonHisp}$	0.01419	0.01934	0.5615	$\beta_{s,EthnNonHisp}$	0.00429	0.00752	0.5683
$\beta_{r,Handedness}$	0.00071	0.00276	0.7965	$\beta_{s,Handedness}$	-0.00102	0.00107	0.3434
$\beta_{r,Income}$	0.00113	0.00140	0.5507	$\beta_{s,Income}$	0.00119	0.00055	0.0418
$\beta_{r,AlcAbuse}$	0.00474	0.00705	0.5725	$\beta_{s,AlcAbuse}$	-0.00099	0.00274	0.7180
$\beta_{r,AlcDep}$	-0.00163	0.01012	0.8720	$\beta_{s,AlcDep}$	-0.00858	0.00393	0.0418
$\beta_{r,SmokStatus}$	0.00551	0.00769	0.5615	$\beta_{s,SmokStatus}$	6.12E-6	0.00299	0.9984
$\beta_{r,COI\times C}$	0.04333	0.23050	0.8509	$\beta_{s,COI\times C}$	-0.04247	0.03949	0.2822
$\beta_{r,COI\times Eglob}$	0.11370	0.87090	0.8962	$\beta_{s,COI\times Eglob}$	-0.00336	0.08083	0.9669
$\beta_{r,COI\times D}$	0.00033	0.00778	0.9661	$\beta_{s,COI\times D}$	-0.00038	0.01329	0.9771
$\beta_{r,COI\times L}$	0.00113	0.04022	0.9776	$\beta_{s,COI\times L}$	0.00284	0.00559	0.6119
$\beta_{r,COI\times Q}$	-0.06496	0.02938	0.0721	$\beta_{s,COI\times Q}$	**0.11180**	0.00482	**<.0001**

* Adjusted using the adaptive FDR procedure described in (Benjamini, Hochberg et al. 2000).

Appendix S6. Macro for model fitting

```
/*************************************************************************/
/* SAS Macro Syntax for Fitting Mixed Models is:                         */
/*                                                                       */
/* %mixnet (data= ,response= ,COIcovars= ,CONcovars= ,NETcovars= ,       */
/* INTcovars,Bin_REs,Cont_REs,subject),                                  */
/*                                                                       */
/*                                                                       */
/* where "data" specifies the dataset to be used,                        */
/* "response" specifies the Y (outcome) variable,                        */
/* "COIcovars" is a list of the covariates of interest                   */
/* "CONcovars" is a list of the confounding covariates                   */
/* "NETcovars" is a list of the network covariates                       */
/* "INTcovars" is a list of the interaction covariates                   */
/* "Bin_REs " is a list of the random effects for Part I model           */
/* "Cont_REs" is a list of the random effects for Part II model          */
/* "subject" variable that indicates the subject id                      */
/* Orthonormal paolynomials is first added and then glimmix is used to   */
/* fit the statistical models.                                           */
/*************************************************************************/

%macro mixcorr(data1,response,COIcovars,CONcovars,NETcovars,INTcovars,
Bin_REs,Cont_REs,subject);


/* **************************************************** */
/* Adding Orthonormal Polynomials */
proc iml;
  orth_poly = orpol(1:19,18); *19= # of windows;
  window_vec = 1:19;
  orth_mat = window_vec`||orth_poly[,2:19];
  create temp1 from orth_mat;
  append from orth_mat;
  close temp1;
quit;
data two;
  set temp1;
  window_index = col1;
run;

proc sort data=two;
 by window_index;
run;

data data;
  merge data1(in=in01) two(in=in02);
  by window_index;
  if in01 and in02;
  intercept = 1;
  ORPOL_TIME_1 = col2;
  ORPOL_TIME_2 = col3;
  ORPOL_TIME_3 = col4;
  ORPOL_TIME_4 = col5;
  ORPOL_TIME_5 = col6;
```

```
  ORPOL_TIME_6 = col7;
  ORPOL_TIME_7 = col8;
  ORPOL_TIME_8 = col9;
  ORPOL_TIME_9 = col10;
  ORPOL_TIME_10 = col11;
  ORPOL_TIME_11 = col12;
  ORPOL_TIME_12 = col13;
  ORPOL_TIME_13 = col14;
  ORPOL_TIME_14 = col15;
  ORPOL_TIME_15 = col16;
  ORPOL_TIME_16 = col17;
  ORPOL_TIME_17 = col18;
  ORPOL_TIME_18 = col19; /*ONLY HAVE TO CREATE ONCE AND THEN REMOVE ORDERS
FROM MODEL FIT TO ASSESS AIC, DON'T HAVE TO RECREATE WITH FEWER POLYNOMIALS*/
run;

/* ************************************************** */


data data;
 set &data;
 if &response=0 then YN=0;
 else if &response>0 then YN=1;
run;

data data0;
 set &data;
 if &response>0;
run;


/* ************************************************** */

/*Binomial Model*/

title2 'Binomial Model (Uncorrelated)';

proc glimmix data = data; *nobound method=rmpl;
 nloptions maxiter=500;
 class &subject; *DyadInd_35; *dyad;
 model yn (desc)= &COIcovars &CONcovars &NETcovars &INTcovars ORPOL_TIME_1-
ORPOL_TIME_12/dist=binary ddfm=residual solution stdcoef;
 random &Bin_REs/ sub=&subject type=vc; *v; *type=chol;
 random ORPOL_TIME_1-ORPOL_TIME_12/ sub=&subject type=vc;
 *covtest/cl(alpha=0.05 type=plr);
 *lsmeans DyadInd_35;
run;


/*Normal Model*/

title2 'Normal Model (Uncorrelated)';

proc glimmix data = data0; *nobound method=rmpl;
 nloptions maxiter=500;
```

```
 class &subject; *DyadInd_35; *dyad;
 model &response = &COIcovars &CONcovars &NETcovars &INTcovars ORPOL_TIME_1-
ORPOL_TIME_12/dist=n ddfm=residual solution stdcoef;
 random &Cont_REs/ sub=&subject type=vc; *v; *type=chol;
 random ORPOL_TIME_1-ORPOL_TIME_12/ sub=&subject type=vc;
 *covtest/cl(alpha=0.05 type=plr); *3 hours to calculate;
 *lsmeans DyadInd_35;
run;

title;


%mend;

/* ********************************************************************** */
/* ********************************************************************** */
```

Appendix S7. Matlab script for simulating dynamic brain networks

```
% *********************************************************************** %
% ---------------------------------------------------------------------  %
% Matlab Script for Simulating Dynamic Brain Networks from mixed models    %
% This script uses estimated parameters from the mixed models to simulate  %
% dynamic brain networks for 10 dynamic networks from 50 subjects, both    %
% selected randomly. For each dynamic network, 10 networks are simulated.  %
% This script is written in a style to favor simplicity and readability    %
% with respect to detailed simulation steps in the paper.
% ---------------------------------------------------------------------  %
% *********************************************************************** %

% Load estimated parameters for fixed(β)and random(b)effects

% CoefsProbModel: estimated fixed effects parameters from eq.2

% CoefsStrModel: estimated fixed effects parameters from eq.3

% VarProbModel: estimated random effects parameters for network/distance
                covariates from eq.2

% VarStrModel: estimated random effects parameters for network/distance
               covariates from eq.3

% VarProbModelPoly: estimated random effects parameters for orthonormal
                    polynomials from eq.3

% VarStrModelPoly: estimated fixed effects parameters for orthonormal
                   polynomials from eq.3

% VarStrSig: estimated variance for the error term from eq.3

% ***********************************************************************

Nsubjs = 50; % number of subjects (out of 200) to be used in simulations
Ndynnets = 10; % number of random networks (out of 19) per subject to be used
                 in simulations

RandSubjs = randi([1 200],Nsubjs,1)'; % Selecting 50 random subjects
RandDynnets = randi([1 19],[Nsubjs,Ndynnets])'; % Selecting 10 random dynamic
                                                  networks

%load RandSubjs; % 50 by 1 vector: saved 50 subject indices selected
                   randomly
%load RandDynnets % 50 by 10 matrix: saved 10 dynamic networks indices
                    selected randomly for each subject

% ***********************************************************************

for n = 1:50

    k = RandSubjs(n); % subject Id:
```

```
% ----------------------------------
% Categorical variables, including: Education, Race, and Ethnicity for
  subject k

EducSubk = CovFile.Education(k); % Education Level: Three Levels
RaceSubk = CovFile.Race(k);      % Race Group: 6 groups
EthnSubk = CovFile.Ethnicity(k); % Ethnicity Group: 3 Groups

EduExpProb = CoefsProbModel(1,EducSubk)*1; % estimated parameter for
                                             EducSubk

EduExpStr = CoefsStrModel(1,EducSubk_Idx)*1; % estimated parameter for
                                               EducSubk
% ----------------------------------
RaceExpProb = CoefsProbModel(1,RaceSubk)*1; % estimated parameter for
                                              RaceSubk

RaceExpStr = CoefsStrModel(1,RaceSubk)*1; % estimated parameter for
                                            RaceSubk
% ----------------------------------
EthnExpProb = CoefsProbModel(1,EthnSubk)*1; % estimated expression for
                                              EthnSubk

EthnExpStr = CoefsStrModel(1,EthnSubk)*1; % estimated expression for
                                            EthnSubk


% Expressions for Categrocal variables
ExCategProb = EduExpProb + RaceExpProb + EthnExpProb; % Probability Model
ExCategStr = EduExpStr + RaceExpStr + EthnExpStr;     % Strength Model

% *******************************************************************
% *******************************************************************
% Binary and Continuous Variables, including: Fluid Intelligence (COI),
% Gender, Age, BMI, Handedness, Income, DSM4_Alc_Abuse, DSM4_Alc_Dep,
% and Smoking Status

% Extracting all covariates for subject k
ContCovsTotSubk = CovFile.CentContCovs(k,CentContCovsIds);

% Expressions for subject k
ExContProb = CoefsProbModel(1,ContCovsTotSubk)*ContCovsTotSubk;
ExContgStr = CoefsStrModel(1,ContCovsTotSubk)*ContCovsTotSubk;

% *******************************************************************

% Simulating random effects for subject k

% Zeros mean vects for both models
MeanVectZero = zeros(length(VarProbModel),1); % length(VarProbModel) =
                                                length(VarStrModel)

MeanVectZeroPoly = zeros(length(VarProbModelPoly),1);
```

```
% Probability model
% variance - covariance matrices
CovMatProbModel = (diag(VarProbModel));
CovMatProbModelPoly = (diag(VarProbModelPoly));

% estimated coefficients (b values)
CoefsProbRand = mvnrnd(MeanVectZero,CovMatProbModel);
CoefsProbRandPoly = mvnrnd(MeanVectZeroPoly,CovMatProbModelPoly);

% Strength model ------------------
% variance - covariance matrix
CovMatStrModel = (diag(VarStrModel));
CovMatStrModelPoly = (diag(VarStrModelPoly));

% b values for the strength model will be simulated simultaneously with
% simulating the strength values

% ********************************************************************

% Simulating networks for each dynamic network

for q = 1:10

    t = RandDynNets(k,q); % Index for dynamic network q for subject k

    Corr_t_Subk = load(Corr_t_Subk_Path); % Corr_t_Subk_Path: Path to
                                            correlation matrix q of subject k

    DistqSubk = load(DistqSubk_Path); % DistqSubk_Path: Path to distance
                                        matrix q of subject k

    % ------------------------------------------------------------
    % Computing network measures using BCT and in house function
     (wfu_compute_leverage_wei)

    % Clustering coefficient
    Clust_Corr_t_Subk = clustering_coef_wu(Corr_t_Subk);

    % Degree
    Deg_Corr_t_Subk = sum(Corr_t_Subk,2);

    % Leverage centrality
    LevCent_Corr_t_Subk = wfu_compute_leverage_wei([],Corr_t_Subk,[],[]);

    % Global efficiency
    len = size(Corr_t_Subk,1); % len = 268
    temp = (1./(Corr_t_Subk.*(Corr_t_Subk>0)+1.*(Corr_t_Subk==0)))...
              *(Corr_t_Subk~=0);
    funcdist = distance_wei(temp);
    Plength = reshape(harmmean(reshape(funcdist(funcdist>0), ...
                (len-1),len),1),len,1); % Path Length
    Eglob_Corr_t_Subk = 1./Plength;  % Global efficiency

    % Modularity
```

```
[~,QQ] = (modularity_und(Corr_t_Subk));
Modul_Corr_t_Subk = QQ.*ones(len,1);




% ------------------------------------------------------
% Network measures
Net_t_Subk = [Clust_Corr_t_Subk Deg_Corr_t_Subk Eglob_Corr_t_Subk ...
    LevCent_Corr_t_Subk Modul_Corr_t_Subk];

% Interactions with fluid intelligence (COI)
IntNet_t_Subk = (ContCovsTotSubk(k,FluidIntelId)).*Net_t_Subk;

% ------------------------------------------------------
% Orthonormal Polynomials (degrees from 1 - 12)
PolySubk = load(PolySubk_Path); % PolySubk_Path: Path to orthonormal
                                  polynomials of subject k

ExPolySubkProb = CoefsProbModel(1,PolySubk)*PolySubk; % estimated
                                       expression for Polynomials

ExPolySubkStr = CoefsStrModel(1,PolySubk)*PolySubk; % estimated
                                     expression for Polynomials


% ******************************************************************
% Reshape all covariates to produce edge-wise covariates
% - i.e., make corresponding covariates for edges connecting i and
% j for i = 1 to 268 and j = (i+1) to 268. This will yield vectorz
% of size 35778 by 1 ((268*(267-1)/2)) for each covariate
% the ij appended to covariate names above represents reshaped
% covariates for connection ij. All continuous covariates should be
% centered and scaled (for covariates with larger values, including:
% degree, age, bmi, handedness, distance, and fluid intelligence)
% with respect to the data used for probability and strength models
% ******************************************************************

% Simulating dynamic brain networks
% kt is appended to indicate dynamic network t for subject k

for i = 1:268
    for j = (i+1):268

        NetSub_ijkt = Net_t_Subk(i,j);
        IntNetSub_ijkt = IntNet_t_Subk(i,j);
        DistSub_ijkt = [Dist_t_Subk(i,j) Dist_t_Subk(i,j).^2];


        ExNetqSubk_ijkt_Prob = ...
           CoefsProbModel(1,NetSubIds)*NetSub_ijkt;

        ExIntNetqSubk_ijkt_Prob = ...
           CoefsProbModel(1,IntNetSubIds)*IntNetSub_ijkt;
```

```
ExDistqSubk_ijkt_Prob = ...
   CoefsProbModel(1,DistSubIds)*DistSub_ijkt;

ExNetqSubk_ijkt_Str = CoefsStrModel(1,NetSubIds)*NetSub_ijkt;

ExIntNetqSubk_ijkt_Str = ...
   CoefsStrModel(1,IntNetSubIds)*IntNetSub_ijkt;

ExDistqSubk_ijkt_Str = ...
   CoefsStrModel(1,DistSubIds)*DistSub_ijkt;

% -----------------------------------------------------------
% Simulating Adjacency and Weighted connectivity Matrices

% Random Effects Expressions
% RndCovsSubk: Intercept, NetqSubk excluding modularity,
  And NetqSubk
% RndPolySubk: Orthonormal Polynomials

ExRndProb_ijkt = [CoefsProbRand ...
   CoefsProbRandPoly]*RndCovsSubk_ijkt;


% Final fixed and random expressions for probability model
 Ex_Fixed_ijkt_Prob = ExNetqSubk_ijkt_Prob +...
    ExIntNetqSubk_ijkt_Prob + ExDistqSubk_ijkt_Prob + ...
    ExCategProb_ijkt + ExContProb_ijkt + ExPolySubkProb_ijkt;

 Ex_Rnd_ijkt_Prob = ExRndProb_ijkt;


 % Final fixed expressions for strength model
 Ex_Fixed_ijkt_Str = ExNetqSubk_ijkt_Str + ...
    ExIntNetqSubk_ijkt_Str + ExDistqSubk_ijkt_Str + ...
    ExCategStr_ijkt + ExContStr_ijkt + ExPolySubkStr_ijkt;

 % -------------------------------------
 % Probability of edge ijkt existing

 % Log odds of probability for connection ijkt
 Logit_pijkt = Ex_Fixed_ijkt_Prob + Ex_Rnd_ijkt_Prob;

 % probability for connection ijkt
 pijkt = (exp(Logit_pijkt))./(1 + exp(Logit_pijkt));

 % ----------------------------------------
 % Fiserzt Strength mean for edge ijkt
 Sijkt_Mean = Ex_Fixed_ijkt_Str;

 % Fisherzt Strength variance for edge ijkt
 VarCovarRndStr_ijkt = ...
    RndCovsSubk_ijkt'*CovMatStrModel*RndCovsSubk_ijkt + ...
    RndPolySubk_ijkt'*CovMatStrModelPoly*RndPolySubk_ijkt ...
```

```
                    + VarStrSig;



                 % --------------------------------------
                 % Simulating 10 networks for network t of subject k

                 for v = 1:10

                 % ----------
                 % Simulating Binary Number from Binomial Distribution
                 Sim_Aijkt = binornd(1,pijkt);

                 % A: a 5-D matrix to be filled with entire simulated
                   adjacency matrices

                 A(i,j,n,q,v) = Sim_Aijkt;
                 A(j,i,n,q,v) = Sim_Aijkt;

                 % ----------
                 % Simulating strength values
                 FZT_Sijkt = normrnd(Sijkt_Mean,VarCovarRndStr_ijkt);

                  % Applying FZT inverse to get simulated raw strength values
                  Sim_Sijkt = (exp(2*FZT_Sijkt) - 1)./(exp(2*FZT_Sijkt) + 1);

                  % S: A 5-D matrix to be filled with entire simulated
                  strength matrices

                  S(i,j,n,q,v) = Sim_Sijkt;
                  S(i,j,n,q,v) = Sim_Sijkt;

               end

            end

        end

    end

end

% ************************************************************************

% Multiplying simulated adjacency and strength matrices to obtain the
% final simulated weighted matrices

W = A.*S;

% *************************************************************************
% *************************************************************************
```

Appendix S8. Subjects and dynamic networks used in simulation

Table S5. HCP subject identification number and dynamic networks used in simulation

**130316**	10	18	11	7	13	5	16	4	8	15
**145834**	13	19	18	11	5	3	15	2	16	4
**414229**	18	12	6	19	14	8	3	1	5	10
**170934**	14	11	7	5	18	17	16	8	1	12
**713239**	18	13	4	9	5	19	14	1	17	15
**172130**	2	9	11	8	5	17	12	4	6	19
**133827**	2	13	18	16	15	5	8	3	7	12
**135932**	11	3	14	5	4	9	16	15	12	1
**160729**	9	3	17	18	7	10	1	14	2	4
**522434**	19	9	1	14	13	3	4	12	15	11
**151425**	8	12	11	2	1	17	10	4	19	5
**175742**	13	2	17	3	14	9	8	5	1	4
**144731**	16	18	8	12	17	9	1	4	13	14
**729557**	6	4	11	1	13	10	2	18	5	9
**178950**	2	6	12	19	5	4	16	13	8	9
**169949**	4	5	1	11	7	2	13	15	17	10
**597869**	10	11	19	1	9	8	7	14	6	4
**690152**	4	5	12	10	6	14	15	18	1	9
**391748**	1	5	8	12	19	9	16	13	15	7
**529549**	7	16	8	5	10	4	11	9	6	3
**213522**	1	13	2	19	14	15	7	16	10	6
**118225**	8	17	16	11	4	14	6	19	2	13
**149337**	4	9	19	11	2	16	3	15	18	12
**190031**	5	6	18	11	3	7	9	19	13	12
**200109**	10	8	12	6	13	11	15	14	17	3
**541640**	15	4	16	7	10	17	18	19	1	6
**280941**	17	9	3	5	15	13	6	8	2	14
**171330**	6	18	3	4	15	14	7	2	16	10
**193239**	11	5	6	3	17	13	7	19	12	14
**583858**	9	11	3	15	12	1	19	4	6	5
**744553**	1	3	11	10	6	7	19	14	16	9
**732243**	4	7	1	10	16	6	5	11	12	18
**695768**	4	13	17	14	10	5	1	15	9	16
**159138**	12	5	13	9	11	19	8	2	1	14
**688569**	9	6	7	17	8	10	18	3	5	14
**115017**	5	10	14	2	3	6	13	12	7	19
**123420**	17	15	13	4	19	18	5	1	11	12
**179346**	12	3	19	14	13	1	18	10	6	4

**579665**	5	1	3	2	18	7	9	19	6	4
**378756**	14	15	4	8	3	17	13	5	18	1
**118831**	4	5	1	11	18	9	15	14	10	13
**346945**	18	2	15	12	13	1	4	8	6	7
**107422**	17	19	9	18	2	6	3	12	14	4
**192136**	5	14	19	6	8	16	2	17	10	11
**147030**	8	10	19	7	4	14	16	11	15	5
**121618**	8	13	16	2	15	3	14	17	6	11
**136227**	9	18	6	17	15	5	19	7	4	3
**153833**	1	11	2	15	16	6	3	10	14	18
**207426**	1	19	16	17	4	10	9	13	15	6
**201414**	5	13	4	15	9	8	1	6	14	18

Appendix S9. Full Results

As Table S6 shows, among the confounding covariates, spatial distance and square of spatial distance are important covariates in explaining the dynamic patterns of both connection probability and strength, while gender, income, and alcohol dependence are important in explaining the dynamic patterns of just connection strength.

Table S6. Parameter estimates, standard errors, and p-values for dynamic networks

**Probability Model Outputs**				**Strength Model Outputs**			
Parameter	Estimate	SE	*p-value	Parameter	Estimate	SE	*p-value
$\beta_{r,0}$	-0.13770	0.02022	<.0001	$\beta_{s,0}$	0.31190	0.00786	<.0001
$\beta_{r,COI}$	0.00319	0.00295	0.4716	$\beta_{s,COI}$	-0.00086	0.00115	0.4540
$\beta_{r,C}$	-7.22530	0.11490	<.0001	$\beta_{s,C}$	3.07330	0.02008	<.0001
$\beta_{r,Eglob}$	12.5799	0.43460	<.0001	$\beta_{s,Eglob}$	3.86090	0.04105	<.0001
$\beta_{r,D}$	-0.07686	0.00389	<.0001	$\beta_{s,D}$	-0.07111	0.00672	<.0001
$\beta_{r,L}$	0.04332	0.02006	0.0872	$\beta_{s,L}$	-0.21300	0.00273	<.0001
$\beta_{r,Q}$	2.10910	0.01471	<.0001	$\beta_{s,Q}$	-1.35550	0.00241	<.0001
$\beta_{r,dist}$	-0.15460	0.00313	<.0001	$\beta_{s,dist}$	-0.02733	0.00038	<.0001
$\beta_{r,dist2}$	0.07723	0.00156	<.0001	$\beta_{s,dist2}$	0.01615	0.00021	<.0001
$\beta_{r,Gender}$	0.00729	0.00553	0.3749	$\beta_{s,Gender}$	-0.00778	0.00215	0.0005
$\beta_{r,Age}$	0.00538	0.00299	0.1619	$\beta_{s,Age}$	0.00153	0.00116	0.2221
$\beta_{r,EduLev1}$	0.00727	0.01046	0.5173	$\beta_{s,EduLev1}$	0.00269	0.00407	0.5080
$\beta_{r,EduLev2}$	0.00775	0.00748	0.4716	$\beta_{s,EduLev2}$	0.00228	0.00291	0.4329
$\beta_{r,BMI}$	0.00205	0.00277	0.5038	$\beta_{s,BMI}$	0.00076	0.00108	0.4810
$\beta_{r,RaceAm}$	0.00773	0.03843	0.8406	$\beta_{s,RaceAm}$	-0.01823	0.01493	0.2478
$\beta_{r,RaceAsian}$	0.00429	0.01006	0.6696	$\beta_{s,RaceAsian}$	-0.00344	0.00391	0.3788
$\beta_{r,RaceBlack}$	0.00299	0.00892	0.7369	$\beta_{s,RaceBlack}$	0.00711	0.00347	0.0524
$\beta_{r,RacedMore}$	-0.00837	0.02134	0.6949	$\beta_{s,RacedMore}$	-0.00929	0.00829	0.2734
$\beta_{r,RaceUknown}$	-0.00028	0.02041	0.9890	$\beta_{s,RaceUknown}$	-0.00724	0.00794	0.3619
$\beta_{r,EthnHisp}$	0.01896	0.02015	0.4716	$\beta_{s,EthnHisp}$	0.00601	0.00784	0.4429
$\beta_{r,EthnNonHisp}$	0.01536	0.01925	0.4836	$\beta_{s,EthnNonHisp}$	0.00427	0.00748	0.5681
$\beta_{r,Handedness}$	0.00088	0.00274	0.7468	$\beta_{s,Handedness}$	-0.00095	0.00106	0.3713
$\beta_{r,Income}$	0.00116	0.00139	0.4836	$\beta_{s,Income}$	0.00125	0.00054	0.0321
$\beta_{r,AlcAbuse}$	0.00438	0.00702	0.5491	$\beta_{s,AlcAbuse}$	-0.00100	0.00273	0.7141
$\beta_{r,AlcDep}$	-0.00119	0.01008	0.9059	$\beta_{s,AlcDep}$	-0.00881	0.00392	0.0356
$\beta_{r,SmokStatus}$	0.00610	0.00767	0.4836	$\beta_{s,SmokStatus}$	0.00006	0.00298	0.9842
$\beta_{r,COI\times C}$	0.10290	0.11500	0.4836	$\beta_{s,COI\times C}$	-0.02418	0.02009	0.2478
$\beta_{r,COI\times Eglob}$	0.24720	0.43470	0.5696	$\beta_{s,COI\times Eglob}$	-0.00301	0.04106	0.9416
$\beta_{r,COI\times D}$	-0.00140	0.00389	0.7177	$\beta_{s,COI\times D}$	-0.00002	0.00672	0.9978
$\beta_{r,COI\times L}$	-0.01690	0.02006	0.4836	$\beta_{s,COI\times L}$	0.00127	0.00273	0.6423

$\beta_{r,COI\times Q}$	-0.02684	0.01514	0.1619	$\beta_{s,COI\times Q}$	**0.03078**	0.00248	**<.0001**

* Adjusted using the adaptive FDR procedure described in (Benjamini, Hochberg et al. 2000). Bold values show fluid intelligence – related inferential results discussed here.

References

Benjamini, Y., Y. J. J. o. e. Hochberg and B. Statistics (2000). "On the adaptive control of the false discovery rate in multiple testing with independent statistics." **25**(1): 60-83.

Simpson, S. L. and P. J. Laurienti (2015). "A two-part mixed-effects modeling framework for analyzing whole-brain network data." Neuroimage **113**: 310-319.
